# An FGF3-BMP Signaling Axis Regulates Caudal Neural Tube Closure, Neural Crest Specification and Anterior-Posterior Axis Extension

**DOI:** 10.1371/journal.pgen.1006018

**Published:** 2016-05-04

**Authors:** Matthew J. Anderson, Thomas Schimmang, Mark Lewandoski

**Affiliations:** 1 Genetics of Vertebrate Development Section, Cancer and Developmental Biology Lab, National Cancer Institute, National Institutes of Health, Frederick, Maryland, United States of America; 2 Instituto de Biología y Genética Molecular, Universidad de Valladolid y Consejo Superior de Investigaciones Científicas, Valladolid, Spain; University of Oxford, UNITED KINGDOM

## Abstract

During vertebrate axis extension, adjacent tissue layers undergo profound morphological changes: within the neuroepithelium, neural tube closure and neural crest formation are occurring, while within the paraxial mesoderm somites are segmenting from the presomitic mesoderm (PSM). Little is known about the signals between these tissues that regulate their coordinated morphogenesis. Here, we analyze the posterior axis truncation of mouse *Fgf3* null homozygotes and demonstrate that the earliest role of PSM-derived FGF3 is to regulate BMP signals in the adjacent neuroepithelium. FGF3 loss causes elevated BMP signals leading to increased neuroepithelium proliferation, delay in neural tube closure and premature neural crest specification. We demonstrate that elevated BMP4 depletes PSM progenitors *in vitro*, phenocopying the *Fgf3* mutant, suggesting that excessive BMP signals cause the *Fgf3* axis defect. To test this *in vivo* we increased BMP signaling in *Fgf3* mutants by removing one copy of *Noggin*, which encodes a BMP antagonist. In such mutants, all parameters of the *Fgf3* phenotype were exacerbated: neural tube closure delay, premature neural crest specification, and premature axis termination. Conversely, genetically decreasing BMP signaling in *Fgf3* mutants, via loss of BMP receptor activity, alleviates morphological defects. Aberrant apoptosis is observed in the *Fgf3* mutant tailbud. However, we demonstrate that cell death does not cause the *Fgf3* phenotype: blocking apoptosis via deletion of pro-apoptotic genes surprisingly increases all *Fgf3* defects including causing spina bifida. We demonstrate that this counterintuitive consequence of blocking apoptosis is caused by the increased survival of BMP-producing cells in the neuroepithelium. Thus, we show that FGF3 in the caudal vertebrate embryo regulates BMP signaling in the neuroepithelium, which in turn regulates neural tube closure, neural crest specification and axis termination. Uncovering this FGF3-BMP signaling axis is a major advance toward understanding how these tissue layers interact during axis extension with important implications in human disease.

## Author Summary

During embryological development, the vertebrate embryo undergoes profound growth in a head-to-tail direction. During this process, formation of different structures within adjacent tissue layers must occur in a coordinated fashion. Insights into how these adjacent tissues molecularly communicate with each other is essential to understanding both basic embryology and the underlying causes of human birth defects. Mice lacking *Fgf3*, which encodes a secreted signaling factor, have long been known to have premature axis termination, but the underlying mechanism has not been studied until now. Through a series of complex genetic experiments, we show that FGF3 is an essential factor for coordination of neural tube development and axis extension. FGF3 is secreted from the mesodermal layer, which is the major driver of extending the axis, and negatively regulates expression of another class of secreted signaling molecules in the neuroepithelium, BMPs. In the absence of FGF3, excessive BMP signals cause a delay in neural tube closure, premature specification of neural crest cells and negatively affect the mesoderm, causing a premature termination of the embryological axis. Our work suggests that FGF3 may be a player in the complex etiology of the human birth defect, spina bifida, the failure of posterior neural tube closure.

## Introduction

Axis extension in vertebrate embryos requires the coordination of numerous genetic and morphological processes within and between the three germ layers: the endoderm, mesoderm and the ectoderm-derived neuroepithelium. This coordination requires numerous signaling factors for regulation of key events that must occur in a proper sequence.

An example of this is the neuroepithelium, which undergoes morphological movements that transform a sheet of cells into the neural tube. Underlying and driving this essential process are a number of signaling pathways, genetic and epigenetic mechanisms and environmental cues [1–3]. Considering this complexity, it is understandable that neural tube defects (NTDs) are among the most prevalent birth defects in the human population (1:1000) [4] and quite a large number of mouse NTD models (exceeding 240) have been identified [5–7].

Neurulation during vertebrate axis extension is divided into primary and secondary phases. During primary neurulation, the neural tube forms by folding of the neural plate so that the two edges of the plate are spatially near allowing for fusion at the dorsal midline [8]. Closure along the spinal axis initiates at the hindbrain/ spinal cord boundary and proceeds caudally, ending with closure of the posterior neuropore in the trunk region. Failure to close the neuropore results in spina bifida (Latin for “split spine”), which remains the most frequent type of human NTD, despite a decrease in frequency after the mandatory addition of folic acid to enriched grain products in the USA [9]. Spina bifida has a 1-year survival rate of 79 to 96%, depending on race/ethnicity [9]; however, surviving patients often experience lifelong disabilities and mortality increases into young adulthood [10].

Closure of the posterior neuropore marks the end of primary neurulation. Neural tube formation caudal to this region occurs via the process of secondary neurulation, involving a mesenchymal to epithelial transition of cells within the tailbud to generate a tube [11,12]. These cells reside within a region of the tailbud called the “chordoneural hinge” (CNH) that contains not only neural tube progenitors but also cells that generate caudal somites and notochord [13–15].

Concomitant with axis extension and neurulation, is the formation of the neural crest: a transient, migratory lineage that delaminates from the dorsal neural tube and forms various cell types that include the melanocytes, craniofacial cartilage and bone, and peripheral and enteric neurons/glia. The timing of trunk neural crest emigration has been shown to be controlled by caudal FGF signals and rostral retinoic acid signals from the adjacent mesoderm [16].

Human neurocristopathies, syndromes caused by dysregulation of the neural crest, occur as often as NTDs [17,18], and there is a limited overlap of the two types of syndromes [17]. Likewise, there is a subset of mouse mutants where defects occur in both neural tube closure and neural crest development [5]. These include mutations in components of the BMP signaling pathway, demonstrating that the proper regulation of this pathway is essential to both events [19–22]. In the caudal trunk, exogenous BMP will inhibit the necessary morphogenic movements within the neuroepithelium, which are necessary for neuropore closure, while premature closure occurs in *Bmp2* null homozygotes [23]. However, after closure, BMP signaling is required for neural crest delamination from this region. Both BMP4 and BMP7 will induce caudal neural crest cell programs [24] and data from manipulation of the chick embryo support the idea that BMP4 in the dorsal neural tube is required for the delamination of caudal neural crest [25–27]. Thus, dorsal BMP activity in the caudal neural tube must be countered because it impedes neural tube closure but afterwards is required for neural crest development. One way the vertebrate embryo carefully calibrates BMP activity in this region is by a balance between BMPs and their antagonist, NOGGIN [25,26]. Consistent with this idea, *Noggin* null homozygotes display both an open neural tube and excessive neural crest development [23,28–30].

Here we demonstrate that FGF3 signals from the presomitic mesoderm (PSM) provide another level of regulation of caudal neural tube BMP signaling. Multiple FGFs are expressed in the presomitic mesoderm and we have recently demonstrated that both *Fgf4* and *Fgf8* can each encode a classical activity known as the “wavefront” that prevents differentiation of the PSM during somitogenesis [31]. Simultaneous inactivation of both of these *Fgf* genes, at various embryonic timepoints, causes an AP axis truncation [31,32]; however, inactivation of either of these *Fgfs* alone causes no apparent extension defect, demonstrating redundancy of this system [31,33]. Furthermore mouse single gene inactivation studies demonstrate that of the five other *Fgfs* expressed in the PSM (*Fgf3*, *Fgf5*, *Fgf15*, *Fgf17*, and *Fgf18*), only *Fgf3* has a requirement in axis extension (reviewed in [27]). Our studies reveal that the *Fgf3* axis truncation is caused by an upregulation in BMP signals from the caudal neural tube and that these signals also cause an increase in neuroepithelial proliferation, delay in neural tube closure and premature neural crest formation.

## Results

### *Fgf3* expression in the nascent mesoderm is required for normal axis extension

**Fig 1:**
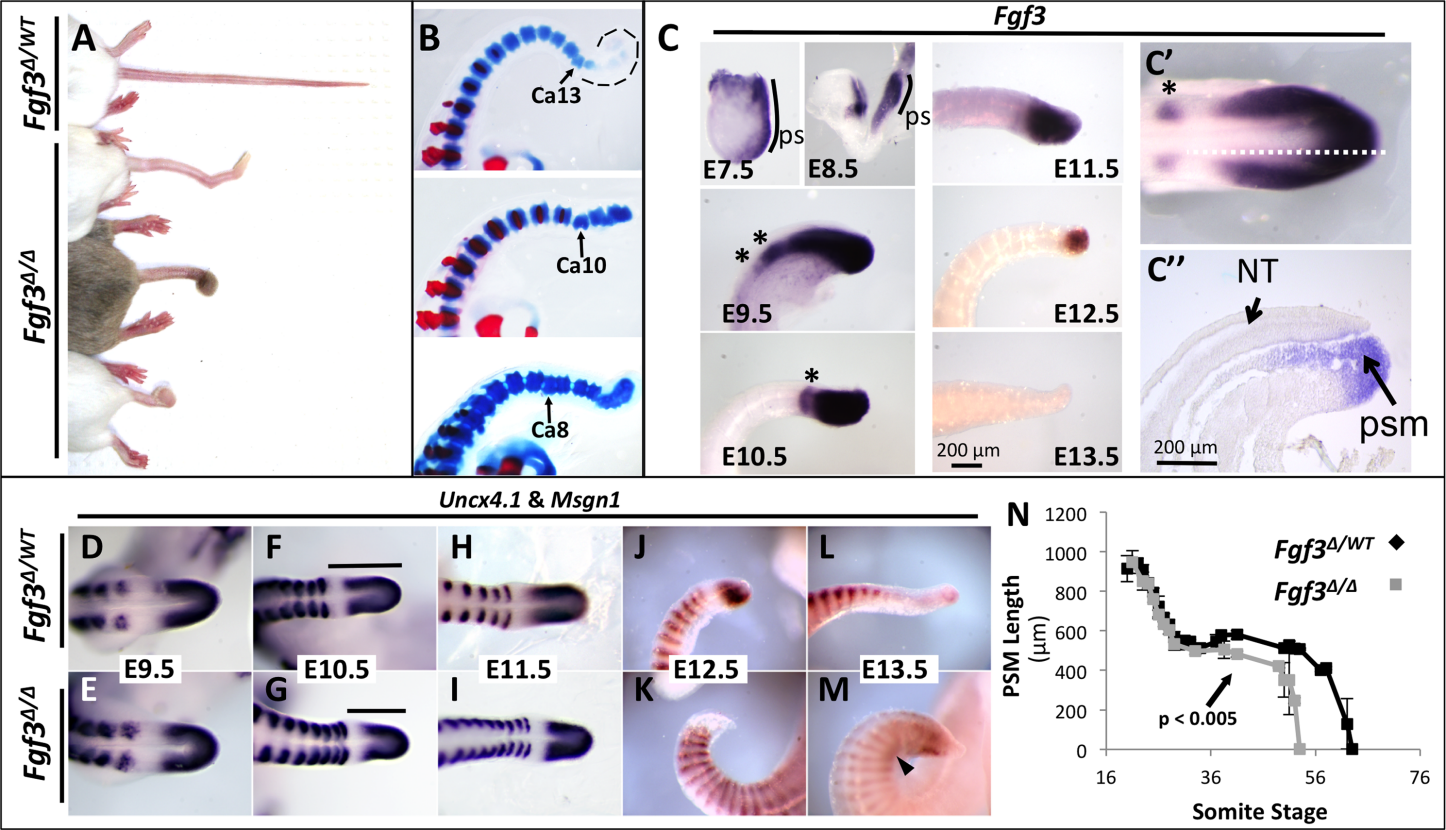
Loss of *Fgf3* causes variable anterior-posterior axis truncation and caudal malformation. (**A**) Dorsal view of three *Fgf3* mutants (*Fgf3*^*Δ/Δ*^) and one littermate *Fgf3* heterozygous (*Fgf3* ^*Δ/WT*^) control showing a variably truncated and sometimes “knotted” tail (bottom two samples) in mutants. (**B**) Skeletal preparations of three E18.5 *Fgf3* mutants demonstrating the variable position of the first vertebral defect (**arrows**; **Ca**, caudal vertebrae. **Dotted lines** outline unstained cartilage). (**C**) Wholemount *in situ* hybridization (WISH) assays for *Fgf3* at the stage indicated. *Fgf3* mRNA is detected in the primitive streak (**ps**, E7.5, E8.5) as well as in the presomitic mesoderm (E8.5–12.5) and tail bud (E9.5–12.5). Expression is absent at E13.5. An apparent dynamic domain of *Fgf3* is observed in the anterior PSM (**asterisks**) (see S1 Fig). *Fgf3* expression is confined to mesoderm and absent in the posterior neural tube (**C”**, with approximate plane of section shown by dotted line in **C’**. **NT**, neural tube; **psm**, presomitic mesoderm, 24 ss); E7.5-E13.5 images taken at the same magnification (**scale bar, E13.5**); images in C’ and C” taken at the same magnification (**scale bar, C”**). (**D-M**) WISH assay for *Uncx4*.*1*, which stains the somites, and *Msgn1*, which stains the caudal PSM, in mutants and littermate controls at the stages indicated. Caudal PSM (*Msgn1* domain) is depleted at E12.5 in mutants and E13.5 in controls. **D-I** are dorsal views; **J-M** are lateral views. Arrowhead in **M** indicates touching *Uncx4*.*1* domains. Bars in **F** and **G** indicate total PSM length, which is quantified in **N**. Note that a decrease in the amount of mutant PSM begins at 37 ss followed by a total loss of PSM around 54 ss, resulting with fewer somites in mutants. Error bars represent SEM; significance determined by two-tailed t-test.

*Fgf3*^*Δ/Δ*^ (“Δ” = deletion and null) mice used in this study [34] have a shortened tail (Fig 1A) similar to that in mice homozygous for the first *Fgf3* null allele generated by Capecchi and coworkers [35,36]. This defect is 100% penetrant, with variable expressivity in two parameters: the total tail length (Fig 1A) and the axial position of the anterior-most (and therefore the earliest to develop) malformed vertebra (Fig 1B). To exclude the possibility that genetic background may affect these parameters, in all genetic experiments described below, we always used littermates for control genotypes (Table 1).

**Table 1:** Experimental crosses used in this study.

Experimental Cross	Experimental Genotype (Frequency)	Control Genotype (Frequency)
***Fgf3***^***WT/Δ***^ **X *Fgf3***^***Δ/Δ***^	***Fgf3***^***Δ/Δ***^ **(1/2)**	***Fgf3***^***WT/Δ***^ **(1/2)**
***Fgf3***^***Δ/Δ***^***; Bak***^***WT/Δ***^***; Bax***^***WT/Δ***^ **X *Fgf3***^***Δ/Δ***^***; Bak***^***WT/Δ***^***; Bax***^***WT/Δ***^	***Fgf3***^***Δ/Δ***^***; Bak***^***Δ/Δ***^***; Bax***^***Δ/Δ***^ **(1/16)**	***Fgf3***^***Δ/Δ***^ **(1/16)**
***Fgf3***^***Δ/Δ***^***; Bak***^***Δ/Δ***^***; Bax***^***WT/Δ***^ **X *Fgf3***^***Δ/Δ***^***; Bak***^***Δ/Δ***^***; Bax***^***WT/Δ***^	***Fgf3***^***Δ/Δ***^***; Bak***^***Δ/Δ***^***; Bax***^***Δ/Δ***^ **(1/4)**	***Fgf3***^***Δ/Δ***^***; Bak***^***Δ/Δ***^ **(1/4)**
***Fgf3***^***Δ/Δ***^ **X *Fgf3***^***Δ/Δ***^***; Nog***^***Lacz/WT***^	***Fgf3***^***Δ/Δ***^***; Nog***^***Lacz/WT***^ **(1/2)**	***Fgf3***^***Δ/Δ***^ **(1/2)**
***Fgf3***^***Δ/Δ***^***; Bmpr1b***^WT/Δ^*X *Fgf3*^***Δ/Δ***^***; Bmpr1b***^WT/Δ^*	***Fgf3***^***Δ/Δ***^***; Bmpr1b***^Δ/Δ^*(1/4)	***Fgf3***^Δ/Δ^*(1/4)

Note: all “Δ” (deletion) alleles are null alleles.

* Animals also contain Bmpr1a ^flox/flox;^ Rosa26 ^mtmg/mtmg^; see Methods and Materials

To understand the *Fgf3* phenotype, we first considered where the gene is expressed. *Fgf3* transcripts have been detected in the nascent mesoderm starting in the primitive streak at E7.5 through E9.5 [37], before closure of the posterior neuropore around E10.0 [38]. We confirmed and extended these analyses and found that *Fgf3* expression is maintained in the nascent mesoderm, emerging from the primitive streak or tail bud, throughout development until axis extension ceases at E13.5 (Fig 1C); *Fgf3* expression is restricted to the tail bud mesoderm and not found in the adjacent neuroectoderm (Fig 1C’ and 1C”). We also noted that an *Fgf3* expression domain exists in the anterior presomitic mesoderm (PSM) that apparently oscillates because its dimensions varied between embryos of the same embryonic stage (asterisks in Fig 1C and 1C’; S1 Fig).

Given this oscillatory expression domain and the work we and others have published demonstrating that different FGFs play essential roles controlling oscillatory activities during somitogenesis [31,32,39,40], we considered whether the *Fgf3* axis defect might be due to dysregulation of normal gene oscillations in the caudal embryonic axis. To address this, we examined *Hes7* expression, a key clock component that responds to FGF signals [31,41,42], in mutant and control embryos from E10.5 to E10.75. We found that *Hes7* expression oscillates in *Fgf3* null homozygotes in an approximately normal pattern although both the caudal somites and the PSM were abnormally shorter in the anterior-posterior axis (S2 Fig).

We examined this size reduction in the mutant PSM and somites in more detail by simultaneously determining the expression of both *Uncx4*.*1*, which marks the posterior half of each somite [43] and *Msgn1*, which marks the posterior PSM [44]. The total PSM is the area between *Uncx4*.*1* expression in the caudal-most somite and the posterior limit of *Msgn1* expression (black bars in Fig 1F and 1G). Furthermore, the *Msgn1* expression domain is a readout of wavefront activity [45,46] and as such, regresses as the posterior axis extends, eventually dwindling in the growing caudal axis and then disappearing as axis extension ceases [47]. In the developmental time window we examined (E9.5- E13.5) control PSM length is highest at ~20 somite stage (ss) (about E9.25), rapidly decreases till ~28 ss (about E10.0), and then remains relatively unchanged for the next 2 days (Fig 1D, 1F, 1H and 1N). Then on day E12.5 the amount of PSM declines rapidly so that by E13.5 no *Msgn1* expression can be observed because all PSM has been incorporated into somites, forming a total of ~64 somites (Fig 1J, 1L and 1N). These changes in PSM length resemble those originally described by Tam [48]. *Fgf3* mutants have comparable amounts of PSM from 20 ss (~E9.5) (compare Fig 1D and 1E) up to 37 ss (~E10.5) when they possess significantly less PSM (Fig 1N and compare Fig 1F and 1G) and at E12.5 lose all PSM, a full day earlier than controls (Fig 1K). Because of this aberrantly early PSM loss, in this genetic background, *Fgf3* mutants form an average of ~10 fewer somites than control littermates (Fig 1N). The size and shape of the *Fgf3* mutant caudal somites are also clearly smaller at E11.5 (compare Fig 1H and 1I). A consistent dorsal curve of the axis causes the dorsal distance between somites to be shorter than the ventral distance (Fig 1K and 1M) and in some cases, the two *Uncx4*.*1* domains are fused (arrowhead in Fig 1M). These changes in mutant somite shape and number sufficiently account for the final vertebral defects observed in adults (Fig 1A and 1B).

We explored whether these changes in somite morphology were preceded by changes in dorsal-ventral (DV) somite patterning by examining expression of *Pax1* and *Pax3*, [49,50]. This analysis revealed that DV patterning was not affected in *Fgf3* mutants (S3 Fig). Another consideration is that the simultaneous loss of both *Fgf4* and *Fgf8* expression in the PSM affects axis extension [31,32]. We found that expression of neither of these *Fgf* genes was affected at stages preceding PSM loss (S4 Fig), however at stages when the PSM is reduced we did observe a similar loss of *Fgf8* expression as a consequence of tissue loss (inset C, D S4 Fig). In addition, expression of *Spry2* and *Etv4*, two “FGF-target” genes responsive to *Fgf4* and *Fgf8* [31,32], was also not affected (S4 Fig). Thus FGF3 is not necessary for *Spry2* and *Etv4* expression, an observation that builds on our previous insight that FGF3 was not sufficient, when *Fgf4* and *Fgf8* were inactivated, for the expression of these FGF target genes [31]. Therefore *Fgf3* is uniquely required for caudal axis extension, independent of *Fgf4* and *Fgf8* function.

### *Fgf3* mutants display aberrant cell death in the tail bud region

**Fig 2:**
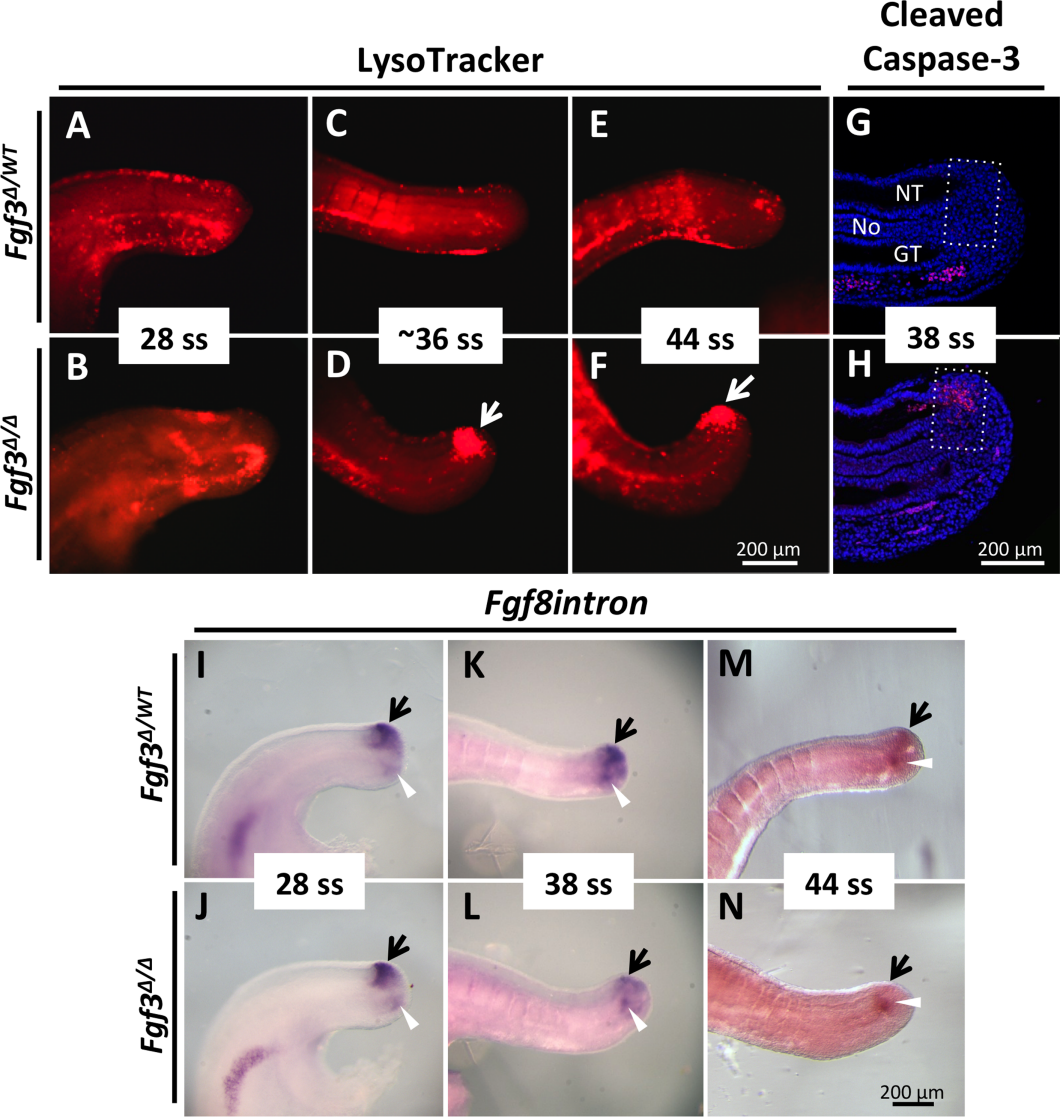
*Fgf3* mutants have aberrant cell death of tailbud progenitors. LysoTracker Red staining of *Fgf3* mutants (**B**, **D**, **F**) and littermate *Fgf3* heterozygous controls (**A**, **C**, **E**), showing a domain of cell death unique to the mutant tails beginning at 36 ss (E10.5) (**white arrow**, **D**) and continuing through 44 ss (E11.5) (**F**); all images taken at the same magnification (**scale bar, F**). Cleaved caspase-3 staining (red) on a sagittally sectioned 38 ss (E10.5) mutant tailbud showing aberrant cell death in the chordoneural hinge region (**G**, **H**; **box** indicates chordoneural hinge region, **NT**: neural tube, **No**: notochord, **GT**: gut tube); images taken at the same magnification (**scale bar, H**). (**I-N**) WISH assay using a riboprobe specific to the nascent *Fgf8* mRNA: *Fgf8intron*, marking the tailbud progenitor domain (**black arrows**), which is similar between *Fgf3* heterozygous controls and *Fgf3* mutants at 28 ss (E9.5) (compare **J** to **I**), diminished in *Fgf3* mutants beginning at 38 ss (E10.5) (compare L to **K**) and gone in *Fgf3* mutants by 44 ss (E11.5) (compare **N** to **M**). **White arrowheads** in **I-N** indicate a domain of *Fgf8intron* expression at the end of the tailgut that is unchanged, thus functioning as an internal control. All views are lateral; all images taken at the same magnification (**scale bar, N**).

Premature loss of *Fgf3* mutant PSM could be explained by a decrease in cellular proliferation and/or an increase in cell death. We measured the rate of proliferation in the PSM at 28 ss and 34 ss and found there to be no difference between mutants and controls (S5 Fig). To determine if there were any changes in cell death within the PSM we stained with LysoTracker Red, which marks domains of cell death [51], in 28 ss (E9.5), 36 ss (E10.5), and 44 ss (E11.5) mutant and control embryos. At 28 ss mutants and control littermates had a similar pattern of staining with a scattering of LysoTracker Red-positive cells in the dorsal neural tube and surrounding caudal end of the tail gut (Fig 2A and 2B). However, beginning around the 36 ss and continuing through 44 ss, an aberrant domain of cell death is observed in the dorsal tail bud caudal to the neural tube (Compare Fig 2D with 2C and 2F with 2E).

To more precisely locate this region of aberrant cell death we immunostained sagittally sectioned mutants for cleaved-Caspase3 [52] and found that the aberrant cell death domain occurs in the CNH region, where progenitors reside that give rise to the mesoderm during secondary axis extension [13–15]. To examine the state of these progenitors, we took advantage of a unique quality of *Fgf8* expression that allows us to specifically label these cells. Although *Fgf8* mRNA is found throughout the PSM, transcription only occurs in the tailbud progenitors that generate the PSM (S6 Fig). As new mesoderm is formed from the progenitors, *Fgf8* mRNA perdures in the daughter cells, as they are displaced from this region and populate the PSM. Decay of mature *Fgf8* transcripts then generates the observed *Fgf8* mRNA expression gradient [53]. This observation demonstrates that the cells actively transcribing *Fgf8* are the progenitors of the PSM. Hence, whereas an exonic riboprobe marks both the PSM and its progenitors, an intronic probe (*Fgf8intron*) can be used to label only the progenitors that are located within the CNH (S6 Fig).

At 28 ss, prior to aberrant cell death, no difference was observed in the intronic *Fgf8* staining domain between *Fgf3* homozygotes and littermate controls (Fig 2J and 2I). However, by 38 ss, the *Fgf8intron* domain in mutants was reduced in the dorsal posterior tailbud (compare Fig 2L with 2K) and was absent by 44 ss (compare Fig 2N with 2M), a day before the PSM is prematurely exhausted (Fig 1K and 1N). These data led us to initially hypothesize that *Fgf3* mutants were losing PSM progenitors, and therefore PSM, due to aberrant cell death.

It is important to distinguish between the loss of the *Fgf8intron* signal that is the result of the *Fgf3* defect and reflects loss of PSM progenitors and the possible contribution of the loss of FGF8 activity to the *Fgf3* axis defect. As mentioned above, analysis of *Fgf8* exon staining and downstream FGF8 target genes indicates no loss of *Fgf8* expression except that due to loss of the PSM tissue (S4 Fig). This suggests no contribution of *Fgf8* loss to the *Fgf3* defect. To test this directly, we used TCre [31,33] to inactivate *Fgf8* in the gastrula mesoderm in an *Fgf3* null background and found that this early inactivation of *Fgf8* had no effect on the *Fgf3* defect (average number of caudal vertebrae: 20.9 for TCre; *Fgf8*^*flox/wt*^*; Fgf3*^*Δ/Δ*^, n = 7 vs. 22.0 for TCre; *Fgf8*^*flox/Δ*^*; Fgf3*^*Δ/Δ*^, n = 6).

### Restoration of cell survival in *Fgf3* mutants exacerbates the mutant phenotype and reveals a neural tube defect

To determine if aberrant cell death was responsible for premature progenitor loss and hence *Fgf3* caudal defects, we sought to restore cell survival by generating *Fgf3* mutants that also lacked *Bak* and *Bax*, which encode pro-apoptotic BCL-2 family members [54–56]. We scored the two parameters of the *Fgf3* defect that we described above in Fig 1A and 1B: 1) the first vertebral body (that is, the most anterior) to be defective and 2) the total number of caudal vertebrae. To exclude genetic background as a factor that might affect variability in these parameters, we intercrossed *Fgf3*^*Δ/Δ*^*; Bak*^*Δ/wt*^*; Bax*^*Δ/wt*^ mice to produce progeny that were triple null for all three loci as well as controls that were only *Fgf3* null homozygotes (Table 1).

**Fig 3:**
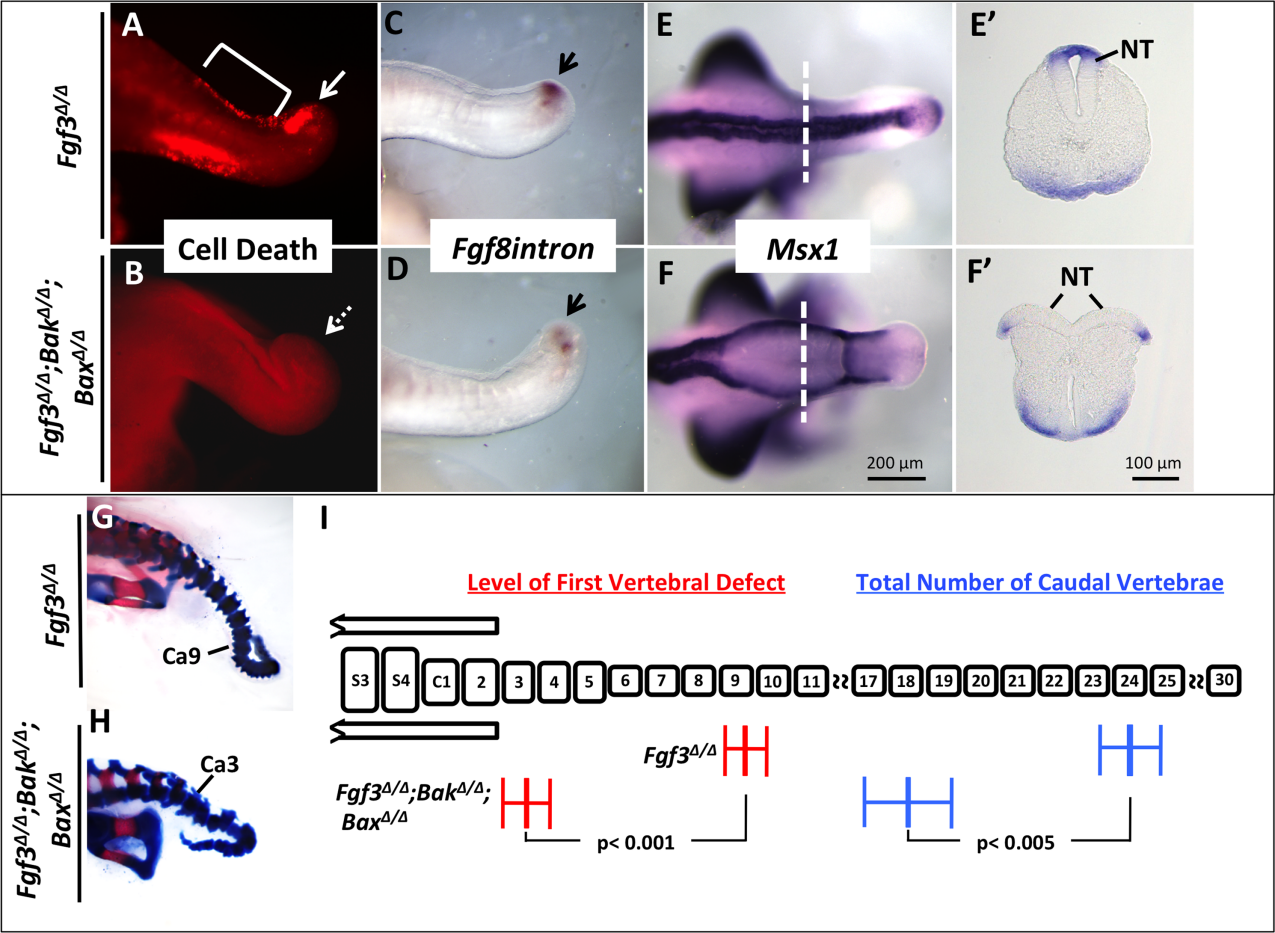
Blocking cell death increases severity of *Fgf3* mutant malformation. LysoTracker Red staining of *Fgf3* ^*Δ/Δ*^*; Bak* ^*Δ/Δ*^*; Bax* ^*Δ/Δ*^ (“triple”) mutants (**B**) and littermate *Fgf3* ^*Δ/Δ*^ embryos (**A**), show a rescue of aberrant cell death in triple mutants (**solid arrow** in **A** indicates domain of cell death and **dotted arrow** in **B** shows domain of restored cell survival, **bracket** in **A** indicates normal domain of cell death in the dorsal neural tube). WISH assays for the markers indicated (**C-F**). *Fgf8intron* expression showing a more severe loss of tailbud progenitors (**black arrows, D**) in triple mutants compared to controls (**C**). (**F, F’**) Dorsal view and transverse section showing a lack of neural tube closure in triple mutants (n = 3/5; compare to control (**E, E’**). Relative position of sections in E’ and F’ indicated by **dotted-white lines in E and F**. Note that *Msx1* was stained to saturation. Skeletal preparations of triple mutants (**H**) and littermate *Fgf3* mutants (**G**) at E18.5 demonstrating that triple mutants have an anterior shift of the first vertebral defect (**Ca**, caudal vertebrae) and a more severe truncation, quantified in **I.** (**I**) Histogram modeling the caudal vertebral column: elements labeled “**S**” or “**C**” represent sacral and caudal vertebrae, respectively. The AP position of the first vertebral defect (**red**) and total number of vertebral elements (**blue**) are as shown for each genotype. For *Fgf3* ^*Δ/Δ*^*; Bak* ^*Δ/Δ*^*; Bax* ^*Δ/Δ*^ mutants, n = 3 and for *Fgf3*^*Δ/Δ*^ mutants, n = 8. Error bars represent SEM; significance determined by two-tailed t-test. Images A-F are taken at the same magnification (**scale bar, F**) and E’-F’ are taken at the same magnification (**scale bar, F’**).

Mice doubly homozygous null for *Bak* and *Bax* have no detectable cell death in the tailbud and form normal caudal vertebrae (S7 Fig). Cell death is also absent in all tailbud tissues of E10.5 *Fgf3; Bak; Bax* triple null homozygotes, including the dorsal neural tube, gut tube and the aberrant PSM domain (compare Fig 3B, with 3A).

Remarkably, removal of *Bak* and *Bax*, rather than rescuing the *Fgf3* defect, worsened it. The dorsal curve of the posterior axis was more pronounced and occurred more anteriorly in compound mutants at E10.5 (compare Fig 3B with 3A) as did the first vertebral defect at E18.5 (Fig 3I). The PSM progenitor domain, marked by the *Fgf8intron* signal, was further reduced in triple *Fgf3; Bak; Bax* E10.5 mutants (compare Fig 3D with 3C) and, likewise, the total number of caudal vertebrae formed was significantly reduced (Fig 3I). Lastly, *Fgf3; Bak; Bax* triple null homozygotes displayed a new defect not immediately apparent in embryos lacking only *Fgf3*: an abnormally open neuropore (n = 3/5) (compare Fig 3F and 3F’ with 3E and 3E’). Moreover, at E18.5 two of five *Fgf3; Bak; Bax* triple null homozygotes from this cross displayed a failure of complete closure of the spinal arches of the sacral vertebrae (spina bifida occulta).

To generate such mutants at a higher frequency (one in four) we intercrossed *Fgf3*^*Δ/Δ*^*; Bak*^*Δ/Δ*^*; Bax*^*Δ/wt*^ mice (Table 1) and found that of thirty E18.5 triple homozygotes, three had spina bifida (compare S8B with S8A Fig). We then prepared skeletons from twelve such mutants without spina bifida and most (nine) displayed spina bifida occulta (compare S8D and S8F Fig to S8C and S8E Fig). To determine if altered proliferation affected the closure of the neural tube of these mutants, we examined rate of proliferation in E9.5 (26–28 ss) and E10.5 (32–34 ss) triple mutant neural tubes. We noted a trend of greater proliferation at the earlier stages in triple mutants but no difference at the later stages (S5B Fig). Therefore we conclude that an increase in cell number through a lack of cell death, and possibly through increased proliferation, leads to the observed failure of neural tube closure. These defects led us to consider whether there were defects in neural tube closure of simple *Fgf3* mutants.

### The earliest morphological defect in *Fgf3* null homozygotes is a delay in neural tube closure

**Fig 4:**
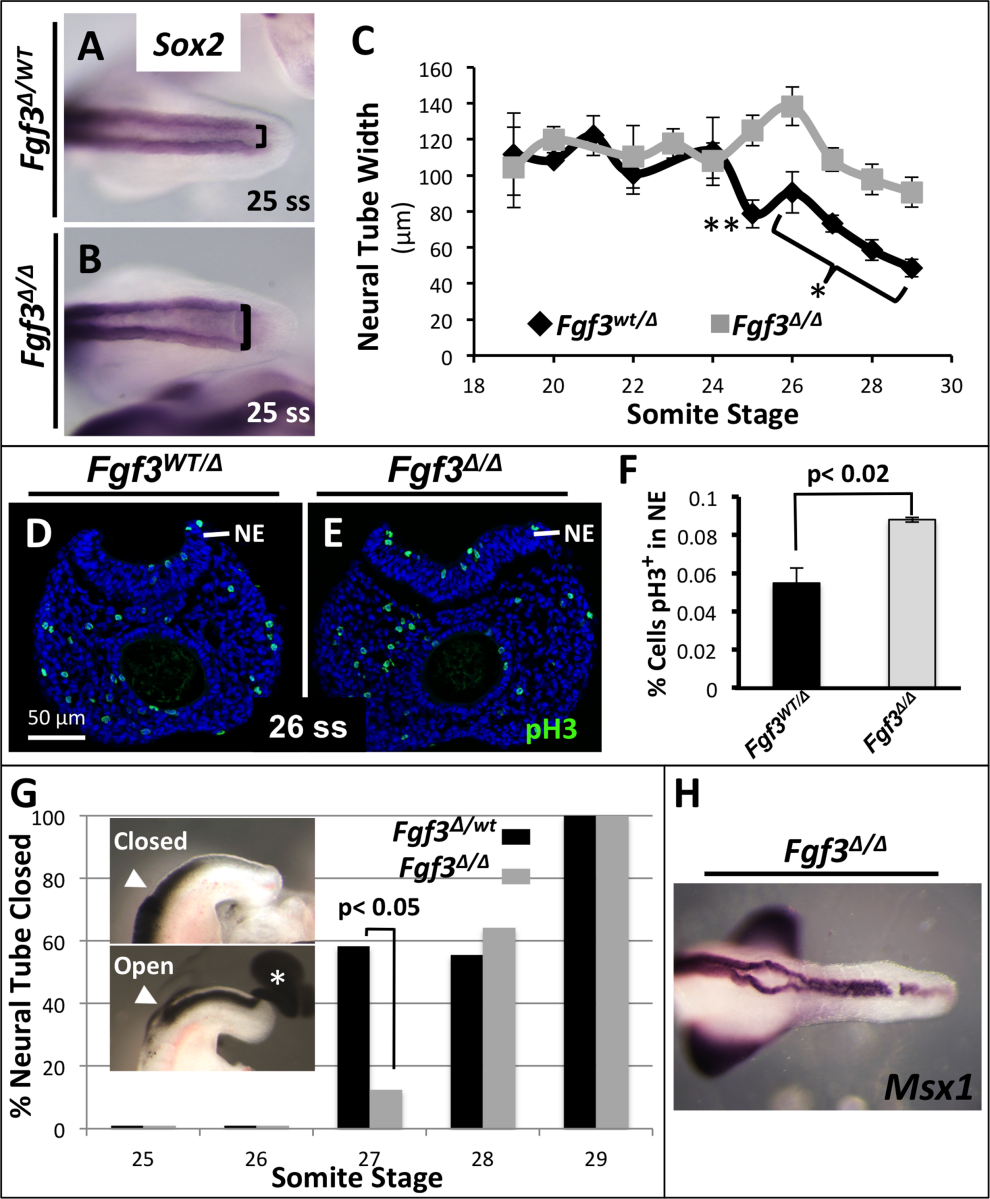
*Fgf3* mutants have defects in neuropore closure. Dorsal view of WISH assay for *Sox2* stained posterior neural tubes showing a widened neuropore in *Fgf3* mutants (**B**, **bracket**) compared to littermate *Fgf3* heterozygous controls (**A**, **bracket**) at 25 ss, quantified in **C**. (**C**) Measurements of posterior neural tube width at various stages demonstrating that the *Fgf3* mutant neural tube is first abnormally widened at the 25 ss (** = p< 0.005, 26–29 ss; * = p< 0.04; significance determined by two-tailed t-test.). Phospho-histone H3 staining showing increased proliferation in mutant neuroectoderm (**E**, compared to control **D**) quantified in **F**. Error bars represent SEM, statistical significance determined by two-tailed t-test (**NE**: neuroepithelium). (**G**) Injection of ink into the neural tube (position of needle insertion indicated by **white arrowheads**) of *Fgf3* heterozygous control and *Fgf3* mutant 25–29 ss embryos reveals a significant delay in closure of mutant neural tube (27 ss) (**asterisk** marks ink from open neuropore). Neural tube closure is complete in all mutants and controls by 29 ss; statistical significance determined by n-1 two proportions test. (**H**) Dorsal view of WISH assay for *Msx1* mRNA (stained to saturation) shows failure of neural tube closure in an *Fgf3* mutant at 10.5.

The original characterization of the axis defect in *Fgf3* null homozygotes found no phenotype in neural tube closure at E11.5, although it was noted that such mutants had an abnormally large neural tube at this stage [36]. We reexamined the *Fgf3* mutant neural tube at earlier developmental stages, motivated by our observations of the severe open NTDs in *Fgf3* ^*Δ/Δ*^*; Bak* ^*Δ/Δ*^*; Bax* ^*Δ/Δ*^ mice (Fig 3F). Prior to 25 ss (E9.5), the width of mutant and littermate-control neural tubes appeared similar (Fig 4C). However, at 25 ss the *Fgf3* homozygous caudal neural tube starts to appear abnormally wide (Fig 4A–4C). We assayed for cell proliferation in *Fgf3* mutants and controls at 26 ss and found that, indeed, *Fgf3* mutants had an increase in the rate of proliferation (Fig 4D–4F). Similar to *Fgf3* ^*Δ/Δ*^*; Bak* ^*Δ/Δ*^*; Bax* ^*Δ/Δ*^ mutants the change in proliferation detected at E9.5 was not observed at E10.5 (32–34 ss, S5 Fig).

To determine exactly when the *Fgf3* mutant neural tube closes, we injected India ink into the neural tube at about the position of the third somite rostral to the PSM; in embryos with an open neuropore, the ink is easily viewed escaping the neural tube lumen (Fig 4G). Such experiments revealed that neuropore closure begins around 27 ss in control embryos but is delayed by about 1 somite stage (2 hours) in the *Fgf3* mutant (Fig 4G). Because this defect is the earliest observed and occurs prior to defects in axis extension (Fig 1), it is the primary *Fgf3* mutant phenotype. As development proceeds, the mutant neural tube remains abnormally large (Fig 4C) but the posterior neuropore closes in most mutant embryos; we have observed occasional individuals with an open neuropore at E10.5 (Fig 4H).

NTDs can be affected by diet and environment [1]. In humans, dietary folic acid (FA) intake during pregnancy has been shown to reduce the occurrence of human NTDs [57]. In mice, the effect of FA supplementation has been explored in twenty-three genetic models to date where it reduced NTD occurrence in seven mutants [58–62], had no effect in twelve mutants [59,61,63–66] and either increased embryonic lethality or induced a more complicated response in four mutants [61,67,68]. To test if the *Fgf3* phenotype is folate-sensitive we crossed *Fgf3* mutant heterozygotes to homozygotes, and intraperitoneally injected pregnant dames at E7.75 with FA. Such injections had no effect on mutant neural tube closure at E9.5 (*Fgf3* heterozygote: average width: 0.882, n = 3; *Fgf3* mutant: average width: 1.887, n = 3). However, at E18.5 only 5/21 (24%) mutants were present instead of the expected 50% (which indeed we observed in uninjected control experiments), suggesting that FA injections caused embryonic lethality of homozygotes. Thus *Fgf3* apparently falls into the gene subset wherein FA treatment causes embryonic lethality of homozygous null mutants [61,67,68].

### BMP signals are increased in the caudal regions of the *Fgf3* mutant neural tube

BMP activity inhibits morphological movements necessary for neural tube closure. *Bmp2*, *Bmp4* and *Bmp7* are all expressed in the dorsal neural tube at the time of posterior neuropore closure with *Bmp4* and *Bmp7* expression occurring rostral to the posterior neuropore [19,23,69]. *Noggin*, which encodes a BMP antagonist, is specifically expressed in the caudal regions of the developing neural tube, inhibiting BMP activity and thus allowing closure of the posterior neuropore [1,70].

**Fig 5:**
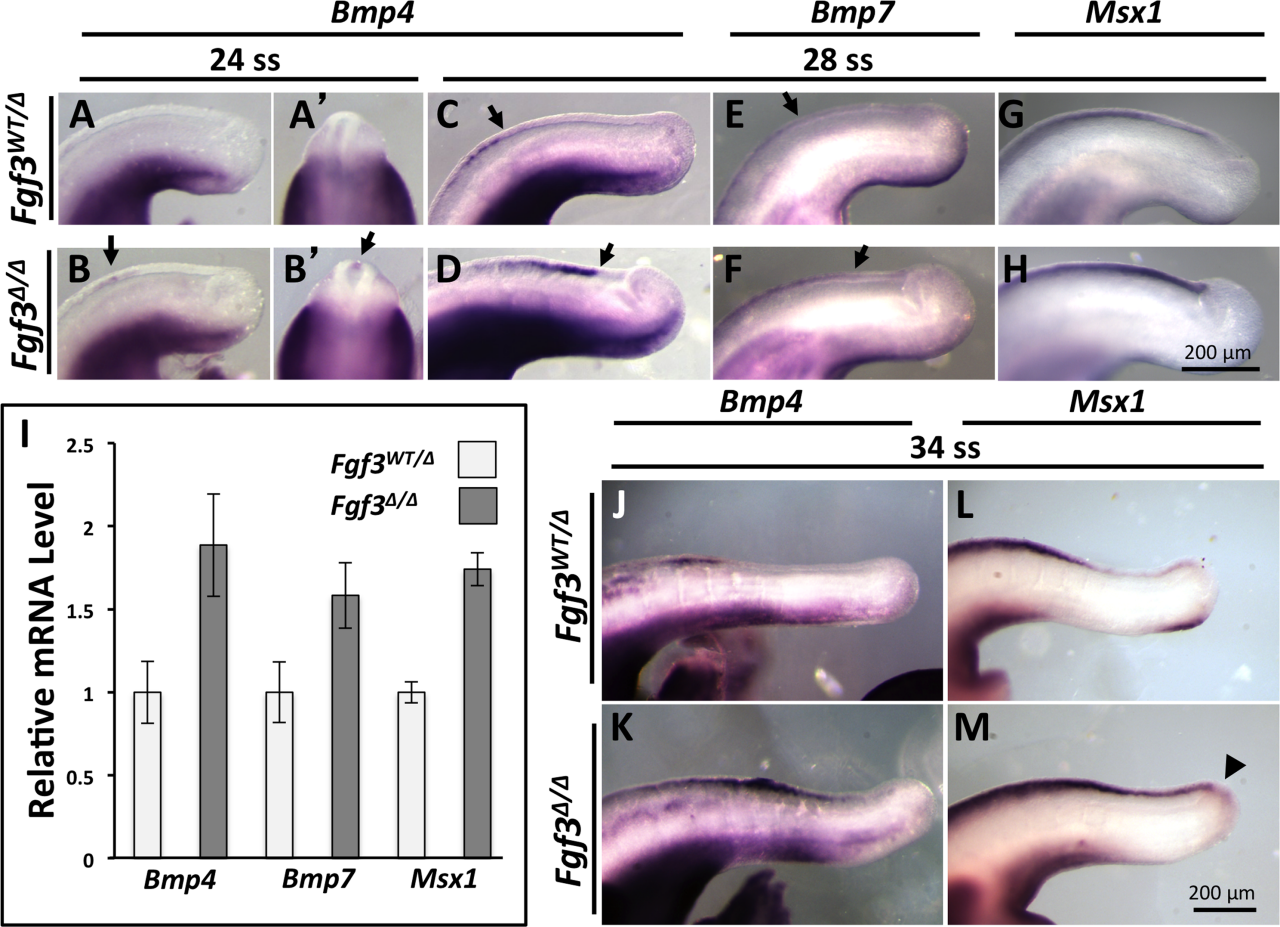
*Fgf3* mutants have upregulated BMP-signaling in the neural tube. Lateral view (**A**, **B**) or dorsal view (**A’**, **B’**, focusing on cross-section of neural tube) of WISH assay reveals that *Bmp4* expression initiates within the dorsal neural tube of *Fgf3* mutants at 24 ss (**B**, **B’**, **arrow**) but not in controls (**A**, **A’**) and is further increased and posteriorly expanded in mutants at 28ss (**D**, **arrows** indicate posterior limit of expression, compare to **C**). (**A-M**) are lateral views of WISH using the probe, genotype and stage indicated. *Bmp7* expression shows a caudal expansion of expression domain in *Fgf3* mutants (**F**, **arrows** indicate posterior limit of expression, compare to **E**). *Msx1* is also upregulated and posteriorly shifted in *Fgf3* mutants (**H**, compare to **G**). Note that *Msx1* WISH was performed for a relatively short period to reveal qualitative differences in intensity between genotypes. (**I**) qPCR using primers specific to *Bmp4*, *Bmp7*, or *Msx1* confirming upregulation in neural tube tissue; error bars represent SEM, graph is representative of at least three experiments. At 34 ss *Bmp4* and *Msx1* expression continues to be caudally expanded in *Fgf3* mutants (**K and M** compare to **J and L**). At this stage *Msx1* expression occurs in the PSM (**arrowhead** in **M**). Images A-H are taken at the same magnification (**scale bar, H**) and J-M are taken at the same magnification (**scale bar, M**).

We found that neural tube-specific *Noggin* expression was unchanged in *Fgf3* mutants starting at 28 ss, when the posterior neuropore is closing, as well as later stages (S9 Fig). However, we found that *Bmp4* expression was initiated prematurely in the *Fgf3* mutant caudal neural tube at 24 ss (Fig 5A–5B’), and remained aberrantly upregulated and extended caudally from 28 ss through 34 ss (Fig 5C, 5D, 5I, 5J and 5K). At 28 ss expression of *Bmp7* was also increased and shifted caudally in mutants (Fig 5E, 5F and 5I). To determine the outcome of BMP activity in mutant neural tubes, we assayed for expression of *Msx1*, a downstream BMP target gene [71–73]. In all *Fgf3* mutants, *Msx1* expression in the dorsal neural tube was upregulated and shifted caudally (compare Fig 5G, 5H, 5I, 5L and 5M).

### Neural Crest is prematurely specified in the caudal regions of the *Fgf3* mutant neural tube

**Fig 6:**
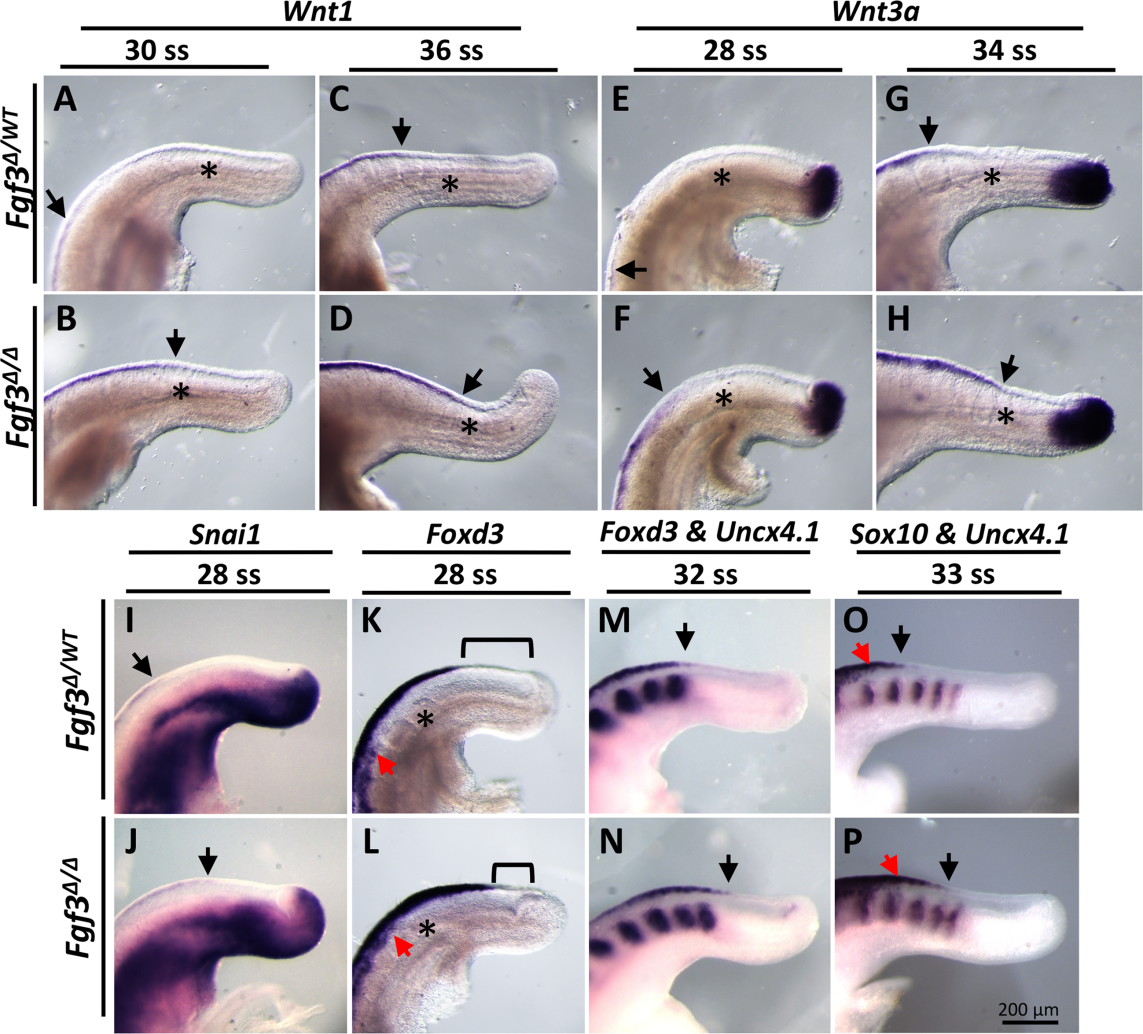
*Fgf3* mutants have caudally expanded neural crest. WISH assay for markers indicated (**A-P,** *Uncx4*.*1* expression marks somites) showing expanded neural crest in *Fgf3* mutants (**B**, **D, F, H, J, L, N, P**) compared to stage matched littermate controls (**A**, **C**, **E**, **G, I, K, M, O**). **Bracket K** and **L** indicates distance between posterior limit of *Foxd3* expression and end of NT; **red arrows** indicate caudal limit of migratory neural crest cells; **black arrows** indicate caudal limit of premigratory neural crest in the dorsal-midline; **asterisks** indicate last formed somite. All images taken at the same magnification (**scale bar, P**).

Neural crest migration within the embryonic trunk is thought to be triggered by BMP4-dependent WNT1 activity [74]. Therefore, based on our observation that BMP activity was precociously upregulated in the absence of *Fgf3*, we asked whether trunk neural crest specification was affected in these mutants. We found that the caudal limit of *Wnt1* expression was extended at 30 ss and remained upregulated through 36 ss (Fig 6A–6D). We also examined *Wnt3a* expression, which is also a marker of caudal neural crest as well as the tailbud PSM [74]. We found *Wnt3a* expression, like *Wnt1*, was notably upregulated in the mutant dorsal neural tube from 28 ss through 34 ss (Fig 6E–6H). Interestingly, we found no change in the PSM domain of *Wnt3a* in these samples, suggesting that altered WNT signals from this region do not cause the *Fgf3* defect (Fig 6E–6H).

To further examine neural crest development in *Fgf3* mutants we assayed for expression of *Snai1* (neural crest specification), *Foxd3* (premigratory and migratory neural crest) and *Sox10* (migratory neural crest) [30,75,76,77]. The caudal limit of *Snai1* expression was extended in mutants, suggesting premature specification of neural crest in the absence of *Fgf3* (Fig 6I and 6J). This idea was confirmed by a caudal extension of both premigratory and migratory neural crest in mutants, as indicated by *Foxd3* and *Sox10* expression at all stages examined (Fig 6K–6P). We then examined the anatomy of caudal dorsal root ganglia, which are derived from the neural crest, in E11.5 mutants by immunostaining for Neurofilament1. This analysis revealed no changes between mutant and littermate controls (S10 Fig). Therefore we conclude that the increase in BMP activity in the caudal dorsal neuroepithelium of *Fgf3* mutants causes a premature specification of neural crest, but without an overall increase in neural crest formation.

### Increased BMP activity causes the axis truncation defect in *Fgf3* mutants

**Fig 7:**
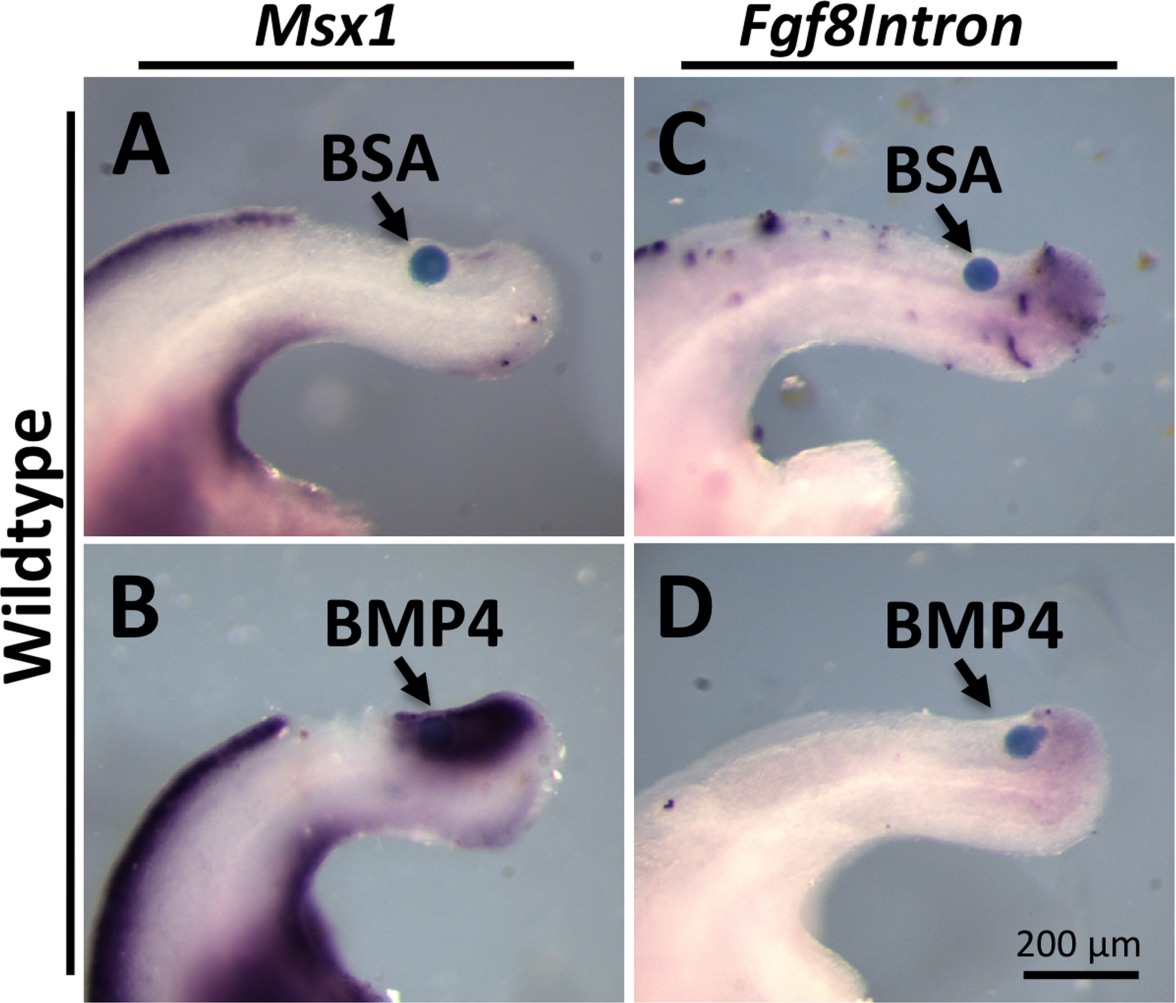
Exogenous BMP reduces PSM progenitors. WISH assay for markers indicated on tail explant cultures with a BSA soaked bead (**A**, **C**) or BMP4 soaked bead (**B**, **D**) inserted into the posterior neural tube of 30 ss wildtype embryos (For **A-D**, n = 4/4, 5/5, 3/4, 5/5 respectively).

We noted that *Msx1* expression in *Fgf3* mutants occurred in the PSM progenitor domain at 34 ss (compare Fig 5M with 5L), suggesting that these cells were receiving BMP signals (a similar *Msx1* expression domain occurs in normal controls, but about a day later, as shown in S11 Fig). Because this is about the time when the mutant PSM progenitor domain begins to diminish (Fig 2L), we asked whether increased BMP signals cause this loss. To test this, we grafted BMP4-soaked beads in the wildtype neural tube just rostral to the progenitor domain and determined that we could readily induce expression of the BMP target gene *Msx1* (compare Fig 7B with 7A). In all such cases (n = 5), we found a loss of *Fgf8intron* signals (compare Fig 7D with 7C), suggesting that increased BMP activity within the progenitors was sufficient for the loss of this population, precipitating shortening of the axis.

To test our hypothesis that an increase in BMP signaling was the cause of these defects *in vivo* we assessed whether they are worsened by increasing BMP signaling or improved by decreasing BMP signaling. We did this genetically, by generating *Fgf3* homozygous embryos and animals that also carried mutant alleles in key BMP signaling genes.

One of these genes is *Noggin* (*Nog*), which encodes the BMP antagonist that plays an essential role in the normal inhibition of BMP signals necessary for posterior neuropore closure. To evaluate the consequences of increased BMP signaling, we generated *Fgf3* null homozygotes that are also heterozygous for a *Nog* null allele (*Nog*^*lacZ/wt*^*)* [28] (Table 1). It is important to note that such *Nog* null heterozygotes are phenotypically normal in a wildtype background.

**Fig 8:**
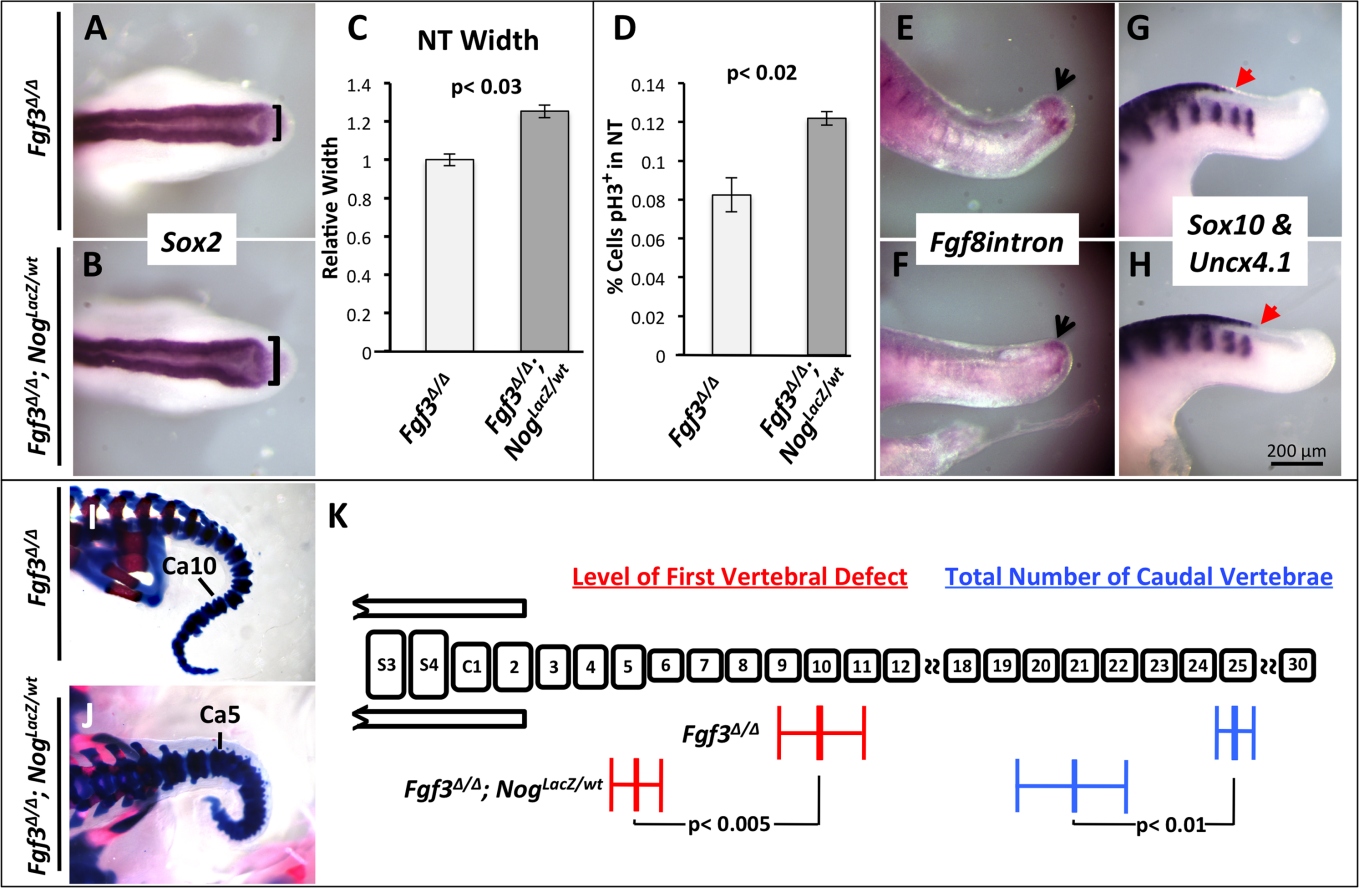
Increased BMP-signaling in *Fgf3* mutants worsens axis truncation. (**A, B, E-H**) WISH assays for probe and genotype shown. (**A, B**) are dorsal views; (**E-H**) are lateral views. *Fgf3*^*Δ/Δ*^*; Nog*^*Lacz/wt*^ mutants have a wider NT at 27 ss compared to *Fgf3*^*Δ/Δ*^ littermate controls, (**B** compare to **A**; **black brackets** indicate measurement of NT width, quantified in **C**), an increase in neuroectoderm proliferation as measured by phospho-histone H3 staining (**D**, 26–27 ss, n = 3 each, percentage represents positive nuclei per total nuclei), a further loss of tailbud progenitors at 37 ss (**F**, compare to **E**, **black arrows** denote tailbud progenitor domain) and a caudal expansion of neural crest at 33 ss (**H** compare to **G; red arrows** indicate posterior limit of expanded neural crest and somites are marked by ***Uncx4*.*1*** expression). Skeletal preparations of E18.5 *Fgf3*^*Δ/Δ*^*; Nog*^*Lacz/wt*^ mutants (**J**) and littermate *Fgf3*^*Δ/Δ*^ mutants (**I**) showing an increase in severity of axis malformation and truncation in the compound mutant (**Ca**: caudal vertebra, marking most anterior vertebral malformation); (**K**) Histogram is similar to that described in Fig 3. For *Fgf3*^*Δ/Δ*^*; Nog*^*Lacz/wt*^ mutants, n = 9 and for *Fgf3*^*Δ/Δ*^ mutants, n = 12. Error bars represent SEM; significance determined by two-tailed t-test. Images in A, B, E–H were taken at the same magnification (**scale bar, H**).

*Msx1* transcripts were more readily detected within the neural tube of *Fgf3*^*Δ/Δ*^*; Nog*^*lacZ/wt*^ embryos, when compared to littermates that were simple *Fgf3* null homozygotes, demonstrating the expected relative increase in BMP signaling (S12 Fig). At 27 ss, these compound mutants, when compared with control *Fgf3* null homozygotes, displayed a neural tube that was on average 35% wider (compare Fig 8B with 8A, quantified in 8C, S12 Fig). This worsening of the neural tube defect is accompanied by an increase in proliferation within the neuroepithelium (Fig 8D). Likewise all other *Fgf3* defects are exacerbated in these compound mutants: the PSM progenitor domain, marked by the *Fgf8intron* signal, is further decreased (compare Fig 8F with 8E) and the caudal limit of neural crest markers is further expanded (compare Fig 8H with 8G). These aggravations of the *Fgf3* null defects in early embryos prefigured changes in later development: in E18.5 *Fgf3*^*Δ/Δ*^*; Nog*^*lacZ/wt*^ embryos, there was an anterior shift in the position of the first defective vertebra as well as an overall reduction in the total number of caudal vertebrae (compare Fig 8J with 8I, quantified 8K). Therefore, increasing BMP signaling increases the severity of the NTDs, neural crest expansion and PSM loss in *Fgf3* mutants.

We then asked the converse question: does reducing BMP signaling improve the *Fgf3* defects? To address this, we generated *Fgf3* null homozygotes that were also lacking *Bmpr1b* [78](Table 1), which is expressed in the developing neural tube [79] and encodes a type-1 BMP-receptor. Mice lacking *Bmpr1b* have no obvious neural tube or axis extension/vertebral defect [80].

**Fig 9:**
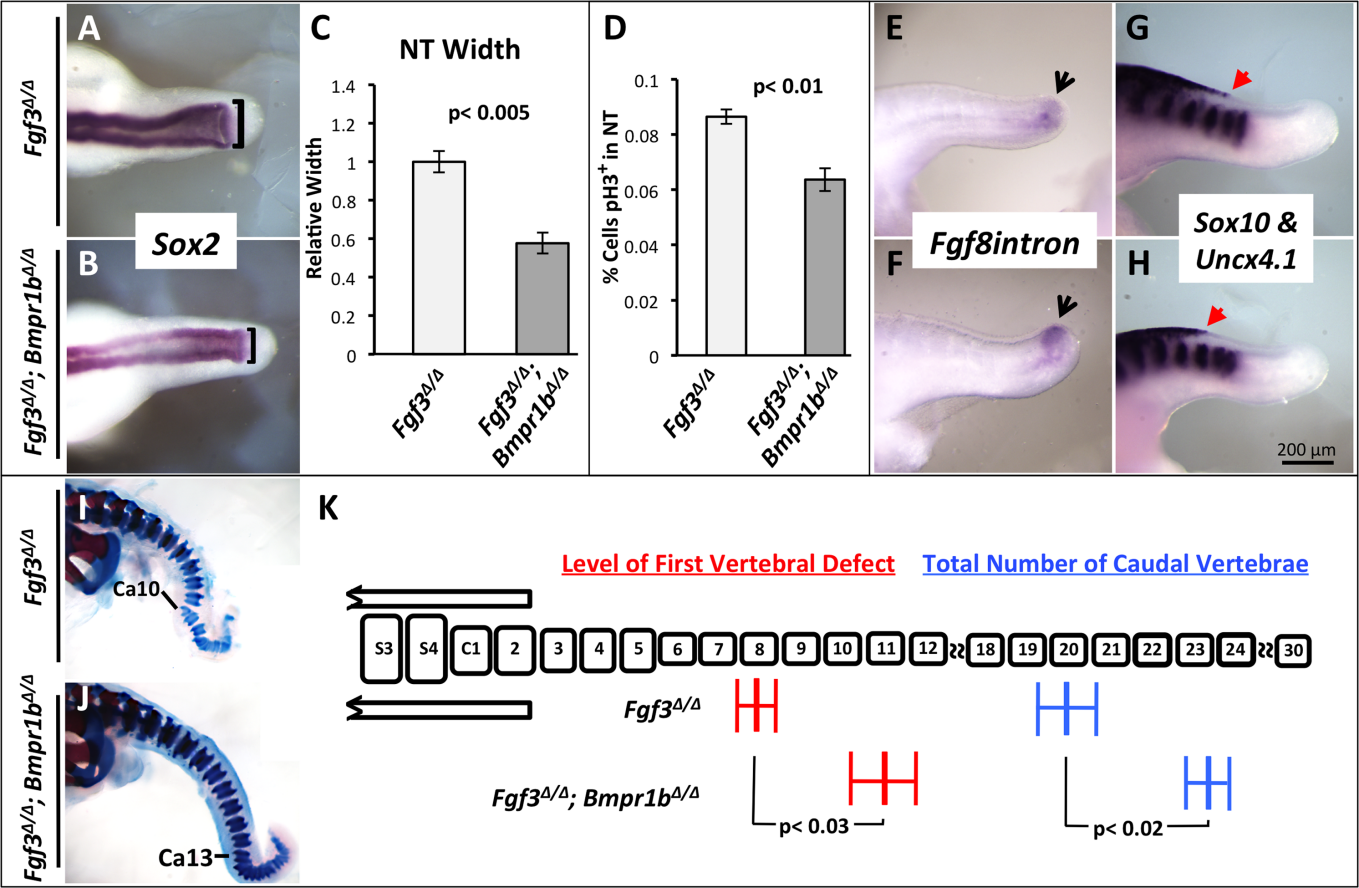
Reduction of BMP-signaling in *Fgf3* mutants partially rescues axis extension. (**A, B, E-H**) WISH assays for probe and genotype shown. (**A, B**) are dorsal views; (**E-H**) are lateral views. *Fgf3*^*Δ/Δ*^*; Bmpr1b*^*Δ/Δ*^ compound mutants, compared to *Fgf3*^*Δ/Δ*^ mutants show a narrower, more normal neural tube at 28 ss (**B** compare to **A**; **black brackets** indicate measurement of NT width, quantified in **C**), a decrease in neuroectoderm proliferation as measured by phospho-histone H3 staining (**D**, 26–27 ss, n = 3 each, percentage represents positive nuclei per total nuclei), an increase in tailbud progenitors (**F** compare to **E**; **black arrows** denote tailbud progenitor domain) and a rostrally shifted, more normal, posterior limit of neural crest cells at 34 ss (**H** compare to **G**; **red arrows** indicate posterior limit of neural crest expansion and somites are marked by ***Uncx4*.1** expression). Skeletal preparations of E18.5 *Fgf3*^*Δ/Δ*^*; Bmpr1b*^*Δ/Δ*^ mutants (**J**) and littermate *Fgf3*^*Δ/Δ*^ mutants (**I**) showing a shift toward normality in both axis malformation and truncation in the compound mutant (**Ca**: caudal vertebra, marking most anterior vertebral malformation); (**K**) Histogram is similar to that described in Fig 3. For *Fgf3*^*Δ/Δ*^*; Bmpr1b*^*Δ/Δ*^ mutants, n = 8 and for *Fgf3*^*Δ/Δ*^ mutants, n = 10. Error bars represent SEM; significance determined by two-tailed t-test. Images in A, B, E–H were taken at the same magnification (**scale bar, H**).

We confirmed that BMP signaling was decreased in *Fgf3*^*Δ/Δ*^; *Bmpr1b*^*Δ/Δ*^ embryos by comparing the intensity of *Msx1* expression in such double mutants to that found in littermates that were null only for *Fgf3* (S12 Fig). At 28 ss double mutants displayed a neural tube that was significantly narrower, and therefore more normal, than that found in *Fgf3* homozygotes (compare Fig 9B with 9A, quantified 9C, S12 Fig) with a corresponding reduction in cellular proliferation within the neuroepithelium (Fig 9D). We observed a definite restoration of the PSM progenitor domain at 34 ss (compare Fig 9F with 9E). Likewise, the caudal limit of neural crest formation was also restored to a more normal level at 34 ss (compare Fig 9H with 9G). At E18.5 we observed a significant shift toward normality in *Fgf3*^*Δ/Δ*^; *Bmpr1b* ^*Δ/Δ*^ mutants in both the position of the first vertebral defect and the total number of caudal vertebrae (compare Fig 9J with 9I, quantified in 9K). Thus, these data support our hypothesis that BMP signals from the neural tube in *Fgf3* null homozygotes cause abnormal axis extension and reducing BMP signaling in these mutants partially rescues all the phenotypic defects.

## Discussion

**Fig 10:**
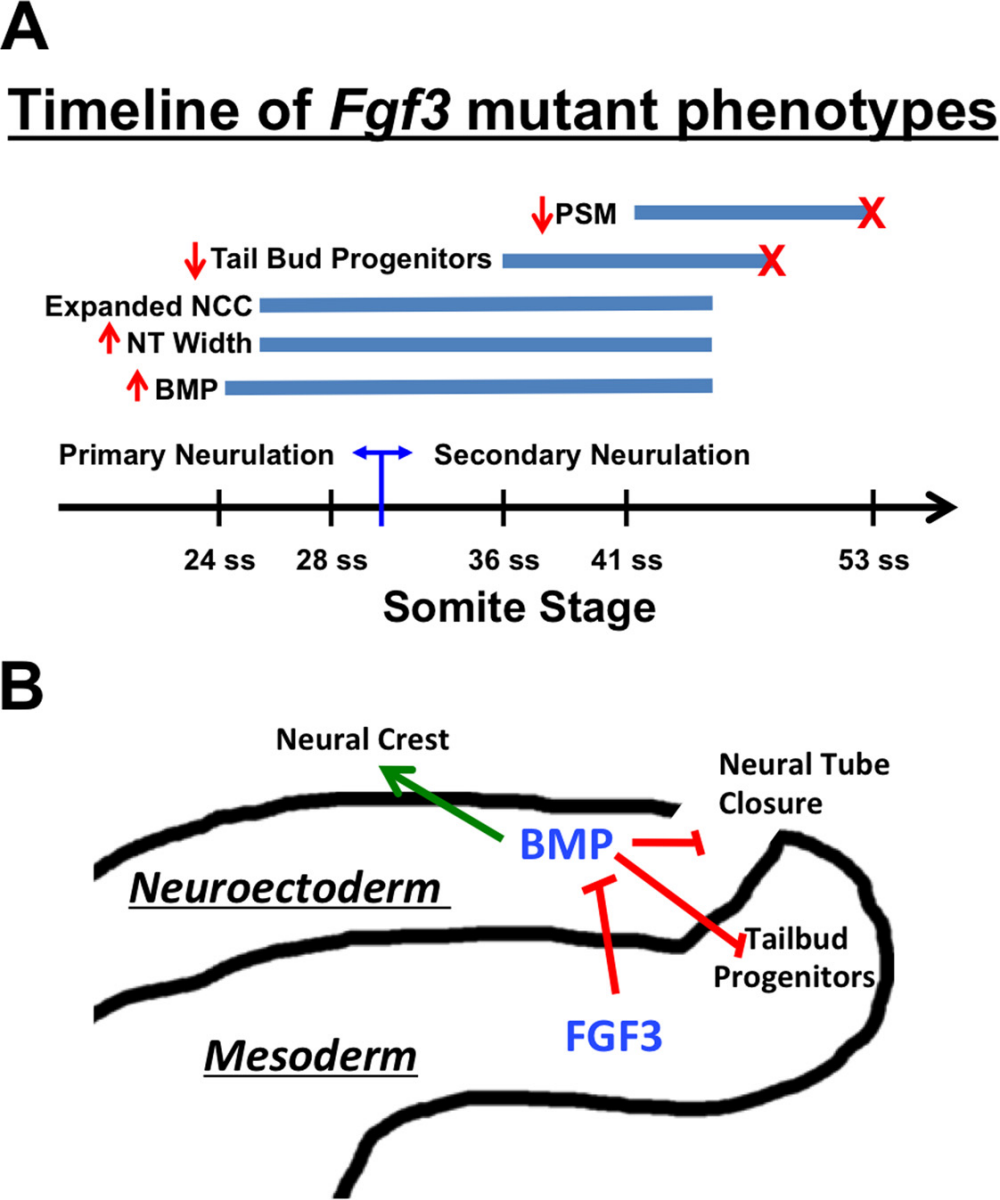
Model and timeline of phenotypes. (**A**) Timeline of phenotypes due to loss of *Fgf3*, indicating onset and duration of observed increased BMP signaling in the neural tube, aberrant neural tube width and caudal neural crest expansion, loss of tailbud progenitors (indicated by *Fgf8intron* staining), and decreased PSM. **Red “X”** indicates premature loss of tailbud progenitors (*Fgf8intron* lost around ~48 ss as opposed to normal loss at >55 ss), and premature loss of PSM. **NCC**, neural crest cells; **NT**, neural tube; **PSM**, presomitic mesoderm. (**B**) Model of FGF3 signaling from the PSM to limit BMP signaling in the neural tube, which positively affects neural crest cell specification, and negatively affects neural tube closure and tailbud progenitors in the mesoderm.

*Fgf3* holds a special place in the history of genetics as the first non-selectable locus to be inactivated in the mouse by gene targeting [81]. Mice lacking *Fgf3* have defects in ear and tail development; although much has been studied about the former phenotype, heretofore little beyond the initial characterization has been analyzed regarding the tail defect [35,36]. Our findings are summarized in Fig 10. We demonstrate that the tail defect is preceded, at E9.5, by premature initiation of BMP signals (both *Bmp4* and *Bmp7*) from the dorsal neural tube. This has two immediate consequences within the neuroepithelium: a delay in closure of the posterior neuropore and abnormal expansion of neural crest during primary neurulation.

Increased *Bmp* expression in the dorsal neural tube can be expected to increase *Wnt* gene expression, and through WNT action, increase both cellular proliferation and neural crest [19,23,26,27,74,82]. Consistent with this, we observe both an increase in *Wnt1* and *Wnt3a* in the caudal neural tube and an increase in proliferation in the *Fgf3* neural tube. Excessive proliferation has been associated with neural tube closure defects [83,84] not surprisingly given the complex patterns of proliferation and cell movement that accompany neural tube closure [85]. When we genetically ratchet BMP signaling up or down, which increases or decreases *Fgf3* neural tube defects, we observe a concomitant increase or decrease, respectively, in proliferation. Hence increased proliferation, through increased BMP activity, may lie at the heart of the *Fgf3* neural tube phenotype.

At E10.5, 24 hours after BMP signals have increased, we observe the first evidence of an axis truncation: loss of PSM progenitor cells (a reduction of the *Fgf8intron* domain) and a shorter PSM (a reduction in the *Msgn1* expression domain). These changes occur during secondary axis formation; thus the *Fgf3* phenotypes straddle primary and secondary neurulation and axis formation. We show that exogenous BMP4 is sufficient to reduce the progenitor domain of the PSM. Furthermore, just as our genetic manipulations of BMP signaling in *Fgf3* mutants affect neural tube phenotypes, they likewise affect axis extension defects, either increasing them when one *Noggin* allele is removed or decreasing them when *Bmpr1b* is inactivated. One way to look at *Fgf3* mutants is that they provide a genetic background that is sensitized to differences in BMP signaling.

### Does FGF3 signal directly to the neural tube?

Although our data supports a model in which FGF3 regulates BMP signals in the neuroepithelium, this regulation could be achieved through FGF3 signaling directly to the neuroepithelium or indirectly through an intermediate. While the current study does not conclusively prove either of these possibilities, we favor the former idea (direct signaling). This is because we find no evidence FGF3 signals to the PSM, regulating another factor that signals to the neural tube, e.g. there is no reduction in the expression of the known FGF-target genes *Spry2* or *Etv4* within the PSM of *Fgf3* mutants (S4 Fig). We have previously published data demonstrating that these FGF target genes are downregulated when *Fgf4* and *Fgf8* are inactivated within the PSM, despite *Fgf3* expression, indicating that FGF3 is not sufficient for their expression [31].

Direct FGF3 signals to the neural tube may occur through FGFR1 or FGFR2, as mutations of either result in neural tube malformations. *Fgfr1* homozygous null development arrests prior to neural tube closure [86,87], but chimeric embryos with contributions of *Fgfr1* null cells in the posterior neural tube displayed spina bifida and kinked tails [88]. Furthermore, embryos that carry FGFR1 mutations that have abrogated signal transduction to the MAPK and PI3K pathways have NTDs [89]. Equally compelling is the case for FGFR2. The IIIb isoform of FGFR2 binds FGF3 and is preferentially expressed in epithelia, such as the neuroepithilium [90]. Embryos homozygous for mutations that delete only this *Fgfr2* isoform have tail defects identical to those in *Fgf3* mutants (neural development was not examined in these studies) [91,92]. Neural tube-specific deletions of these receptor genes are needed to explore whether FGF3 signals directly to the neuroepithelium.

### *Fgf3* signaling and genetic redundancy

Although *Fgf3* expression is continuous in the emerging mesoderm starting at E7.5 (this study and [37]), the earliest consequence of *Fgf3* loss is not observed until 24 ss (about E9.5). Therefore if an earlier FGF3 requirement from this domain exists, it must be redundant with another FGF(s). Genetic studies in the mouse reveal that FGF3 shares function with FGF8 and FGF10 in inner ear development [34,93–95], with FGF10 in cardiovascular development [96] and with FGF8 in pharyngeal morphogenesis [97]. Candidate *Fgf* genes expressed within or near the PSM that may function along with *Fgf3* include *Fgf4*, *Fgf5*, *Fgf8*, *Fgf15*, *Fgf17* and *Fgf18* [98], with *Fgf4* and *Fgf8* arguably being the strongest candidates based on their requirements in axis extension [31,32]. We have recently published that *Fgf3* and *Fgf4* are functionally redundant for mesoderm maintenance in the tailbud during secondary axis formation; however we do not see additional defects in mesoderm or neural tube patterning in the primary axis [99]. In the present work we demonstrate that no functional redundancy exists between *Fgf3* and *Fgf8*.

We propose that *Fgf4* and *Fgf8* are required throughout axis extension for PSM gene expression and maintenance whereas *FgF3* is required during a window during the transition between primary and secondary axis extension for neuroepithelium gene expression and normal morphogenesis through BMP action. In support of this idea, expression of FGF target genes in the PSM fails when *Fgf4* and *Fgf8* are inactivated [31,32], even in the presence of persistent *Fgf3* expression [31,32]. Although Boulet and Capecchi found neural tube defects when *Fgf4* and *Fgf8* were inactivated, it is unclear whether these defects were due to a primary or secondary effect of this phenotype. Furthermore, Boulet and Capecchi found that expression of *Wnt3a*, the only neural crest marker in their study, was not upregulated in the neural tube [31,32], as we observe in *Fgf3* mutants (Fig 6).

### Cell death in neural tube closure and axis extension termination

We discovered the neuropore closure defect in *Fgf3* mutants because it was dramatically exacerbated in embryos lacking the pro-apoptotic factors BAK and BAX in which apoptosis was abolished. In normal development, cells at the neural ridges undergo apoptosis before and after neural tube closure [100–102]. Whether this apoptosis is required for normal neural tube closure is somewhat controversial. Inhibiting apoptosis with the pan-caspase inhibitor, zVAD–fmk, causes a failure of neural tube closure in avians [102] but not in mice [103]. Although mice lacking genes that encode intrinsic apoptotic factors such as *Casp3*, *Casp9* or *Apaf1* have neural tube closure defects [104–106], Copp and coworkers have found that the forebrain and spinal regions of the neural tube close in *Casp3* and *Apaf1* mutants [103]. Conflicting data concerning the requirement for these factors may be explained by sensitivities of these defects to genetic background [104]. Also, the requirements for apoptosis may be subtle; Yamaguchi *et al* demonstrated that when closure occurs in *Apaf1* and *Casp3* mutants, it does so with delayed kinetics [101], which is what we observe in *Fgf3* null homozygotes.

Surprisingly, these defects in *Fgf3* mutant neural tube closure were worsened upon abrogating cell death when we additionally deleted BAK/BAX function. In these *Fgf3; Bak; Bax* triple null mutants, not only was the abnormal PSM cell death prevented, but normal cell death in the dorsal neural tube was also inhibited (Fig 3A, bracket). This may result in retention of neural BMP–expressing cells, which, together with enhanced *Bmp* expression due to a lack of *Fgf3*, causes overall high BMP levels causing the observed severe open NTD. Indeed, we find higher *Bmp4* expression in the dorsal neural tube of *Fgf3; Bak; Bax* triple null homozygotes compared to simple *Fgf3* mutants (S13 Fig). Furthermore, there are increased migratory and premigratory neural crest cells in the caudal neural tube of these compound mutants (S13 Fig), consistent with the hypothesis of an increase of BMP signals; this interesting observation clearly warrants further study.

One question our data raises is whether the BMP-mediated loss of PSM progenitor cells in *Fgf3* mutants is an acceleration of a process that occurs during normal axis termination. Prior to normal termination of the axis, PSM-specific *Fgf3* expression is downregulated (Fig 1C) and BMP signaling increases in the PSM progenitor domain of wildtype embryos (as noted by expression of *Msx1* as shown in S11 Fig). This is consistent with the idea that an FGF3-BMP signaling axis may terminate anterior-posterior extension. Genetic proof of this hypothesis would be the production of mice with longer tails due to the inactivation of gene(s) encoding BMP-signaling components. Although we observe no such effect in *Bmpr1b* null homozygotes (the average number of caudal vertebrae was 30 in both *Bmpr1b*^*Δ/Δ*^, n = 4, and *Bmpr1b*^*wt/wt*^, n = 3), other candidate genes remain to be explored.

In chick and fish embryos, current models of axis termination propose that a downregulation of FGFs and WNTs and rising retinoic acid (RA) levels lead to death of the PSM progenitors [107–111]. However, experiments in the mouse suggest that axis termination occurs in the absence of RA [112]. Furthermore, axis truncations due to PSM-specific inactivation of *Fgfr1* or *Fgf4/Fgf8* occur without changes in RA levels [32,39]. Whatever the signals involved, our data suggest that, at least in the mouse, cell death is not required for PSM progenitor loss, either normally during axis extension or prematurely in *Fgf3* mutants, because we find that if we inhibit apoptosis by *Bak*/*Bax* inactivation, axis termination still occurs in both cases (S7 Fig, Fig 3).

### Potential *FGF3* role in human neural tube closure defects

Characterization of mouse tail mutants have led to insights into human syndromes affecting axis extension. For example, mutations in the T locus, which cause a short tail in mice [113], are associated with spina bifida and vertebral malformations in humans [114,115]. Therefore, we speculate that human *FGF3* mutations may also contribute to abnormal neural tube closure or vertebral malformation. This would be consistent with *Fgf3* targeted mouse alleles, which model defects in human tooth and ear development where *FGF3* mutations cause congenital deafness, microtia, and microdontia. (<http://omim.org/entry/164950?search=fgf3&highlight=fgf3>http://omim.org/entry/164950?search=fgf3&highlight=fgf3).

In support of this idea, a genomewide screen for human NTDs found linkage at regions of chromosome 10 (10q25.3) that include *FGFR2* [116], which, as discussed above, encodes a likely FGF3 receptor. Interestingly, Hillbertz et al suggested that mutations within the region of chromosome 11 (11q13) that contains the closely linked genes, *FGF3*, *FGF4* and *FGF19*, may cause human NTDs based on their finding that a duplication of this region causes the hair ridge and predisposition to dermoid sinus in Ridgeback dogs [117]. Dermoid sinus is caused by an incomplete separation of the neuroepithelium and ectoderm during neural tube closure and, in humans, is often associated with spina bifida occulta [118]. We suggest that *FGF3* may be an important gene in this region required for normal neural tube closure and caudal development.

## Materials and Methods

### Ethics statement

Mice were treated in accordance with the recommendations in the Guide for the Care and Use of Laboratory Animals (National Academies Press; 8th edition). The protocol was approved by the Animal Care and Usage Committee of NCI-Frederick (NIH) (Animal Study Proposal: 11–069).

### Mouse lines

Mice were kept on a mixed background. Crosses were performed as described in Table 1, wherein the female genotype is listed on the left side of the cross and male genotype on the right. Genotyping was performed as previously reported for the following alleles: *Fgf3*^*Δ*^ [34], *Bak*^*Δ*^*; Bax*^*Δ*^ [56], *Noggin*^*Lacz*^ [28], *Bmpr1a*^*flox*^ [119] *Bmpr1b*^*Δ*^ [78], *Rosa26*^*MTMG*^ [120]. One exception is that we used the following primers to detect the *Bax* ^*Δ*^ allele: (5’- CAA CTC CTA CCG CAA GTC CTG G-3’ and 5’- GAA CCC TAG GAC CCC TCC G-3’). All *Fgf3*^*Δ/Δ*^*; BmpR1b*^*Δ/Δ*^ and littermate control mice (Fig 8) also carried *BmpR1a*^*flox/flox*^, which, if unrecombined, has wildtype activity, [119] and *Rosa26*^*MTMG/MTMG*^ [120]. Neither these progeny nor their parents contain a Cre transgene or allele and therefore these alleles had no phenotypic consequence.

### Whole mount *in situ* hybridization, skeletal preparations and cell death analysis

Whole mount *in situ* hybridization was performed as previously described [31]. In all cases, control and experimental embryos were processed in the same tubes throughout the procedure. The *Fgf8intron* probe was constructed using the primers: 5’-GGAATTC ACGCAGTCCTTGCCTTTGCCG-3’ and 5’- GGAATTCATCTGCATGAACAAGAAGG-3’, to amplify an ~700bp product from intron 5 of the *Fgf8* gene. For sectioning, embryos were fixed overnight in 4% paraformaldehyde in PBS then dehydrated into 100% methanol. Embryos were then embedded using the JB-4 Embedding kit as per kit protocol (Polysciences, Inc; Catalog #00226). Blocks were sectioned at a thickness of 6μM and counterstained with a neutral red stain. Skeletal preparations were performed as previously described [121]. LysoTracker Red staining was performed as previously described [122].

### Immunohistochemistry

For immunostaining on sections, embryos were embedded in paraffin and 7μM sections were cut. After dewaxing and rehydration, antigen retrieval was achieved by incubating slides in 0.01M Sodium Citrate pH 6.0 at 95°C (20 min). Each slide was incubated with primary antibodies overnight at 4°C and in secondary antibodies for 1 hour at room temperature and then counterstained with DAPI (ThermoFisher, D21490). Primary antibodies: Cleaved Caspase-3 (1:100, Cell Signaling, #9661); Phospho-Histone H3 (1:200, Cell Signaling, #9706). Secondary antibodies: anti-Mouse-488 (1:250, ThermoFisher, A-11017); anti-Rabbit-594 (1:250, ThermoFisher, A11012). All antibodies were diluted in blocking buffer (1% Goat serum in PBS). Confocal images were taken and Z-stacks were constructed using imageJ software [123].

Phosphohistone H3 positive and negative nuclei were counted from 3 sections from each of 3 embryos for each genotype and averaged. Percentages represent number of positive nuclei divided by total nuclei.

Whole mount immunostaining (Neurofilament1) was performed on embryos fixed with 4% PFA overnight at 4°C, then dehydrated stepwise into methanol and stored at -20°C. Embryos were bleached in Methanol: 30% Hydrogen Peroxide (5:1), rehydrated and blocked in PBSMT (2% dried milk, 0.1% Triton x-100 in PBS) for 2 hours at room temperature. Primary antibody (1:100, DSHB, 2H3 Concentrate) was diluted in PBSMT and incubated overnight at 4°C. Embryos were then washed 6 x 1 hour and incubated overnight at 4°C with secondary anti-mouse antibody-488 (1:250, ThermoFisher, A-11017). Embryos were washed and cleared in 50% glycerol before imaging.

### Quantitative PCR

The neural tubes were cleanly microdissected with one cut at the anterior side of the last formed somite and the posterior cut between the neural tube and posterior mesoderm. Dissected tissue were placed in 25μL of Trizol and stored at -80°C. Following genotyping, cDNA was synthesized (iScript synthesis kit, BioRad, #1708891) from mRNA extracted from 3–4 neural tubes. Sybr green reagent with the following primers were used for qPCR: Bmp4 (ACCAATGGAGCCATTCCGTA, ACGACCATCAGCATTCGGTT), Bmp7 (CTACATGAACGCCACCAACC, CACAGCAGGGCTTGGGTAC), Msx1 (CCGTGGATGCAGAGTCCC, GCTTGCGGTTGGTCTTGTGC), GapDH (TGTAGACCATGTAGTTGAGGTCA, AGGTCGGTGTGAACGGATTTG). Relative quantification was determined using the delta-delta-CT method [124].

### Measurements and statistics

Using the measuring tool within Photoshop, linear measurements were made on images of embryos that were taken at the same magnification; units are in pixels. For all data provided, at least an n = 3 was used unless otherwise noted. Significance was determined using a two-tailed t-test unless otherwise noted in the figure legend.

### Neural tube ink injections

For determination of NT closure, India ink was diluted 1:10 in PBS and injected into the NT at the anterior boundary of the hindlimb buds using a pulled glass capillary needle. The posterior neuropore was scored as open if the ink passed through the pore; otherwise it was scored as closed.

### BMP4 bead and tail explant cultures

Recombinant mouse BMP4 (R&D Systems, 5020-BP-010) was reconstituted in sterile 4mM HCl with 0.1% BSA to 100ug/mL as recommended by manufacturer. Affi-Gel Blue Gel beads (50-100mesh, Bio-Rad;153–7301) were briefly washed with PBS, then soaked in reconstituted BMP4 solution for 1hr at 37°C, then cooled and stored at 4°C and used in experiments for up to 2 weeks. Embryos were harvested from wildtype (NIH-Swiss) animals on the morning of E10.5 in 37°C PBS. Using forceps, the embryos were cut in half at the interlimb region and the rostral halves were discarded. A small puncture was made with forceps in the dorsal NT at the anterior boundary of the PSM and a bead was inserted into the lumen of the NT and pushed posteriorly towards the PSM. Tissues were transferred to a well of a 24-well plate and cultured at 37°C 5%CO_2_ in 250μL of defined media [125]: 0.5mL Penicillin-streptomycin (10,000U/mL; Gibco, 15140–122), 1mL non-essential amino acids (Gibco, 11140–035), 1mL sodium pyruvate (Sigma, S8636), 2mL D-glucose (22.5%), 0.1mL sodium L-ascorbate (0.2g/mL, Sigma, A4034), 1mL calcium lactate hydrate (40mg/mL, Sigma, L4388), 0.1mL D-biotin (0.2mg/mL, Sigma, 47868), vitamin B12 (40μg/mL, Sigma, V6629), 0.1mL PABA (2mg/mL, Sigma, A9878), to 100mL Dulbecco’s Modified Eagle Medium (Gibco, 11995). Following incubation, samples where briefly rinsed with PBS before fixation with 4% paraformaldehyde overnight rocking at 4°C. Samples were then dehydrated into 100% methanol and stored at -20°C until processed for WISH.

### Folic acid injections

For folic acid supplementation, pregnant mothers carrying *Fgf3* heterozygous and null embryos were intraperitoneally injected with 25mg/kg FA at E7.75 and allowed to develop to E9.5.

## Supporting Information

S1 Fig *Fgf3* expression is dynamic in the anterior PSM. WISH assay for *Fgf3* mRNA expression in WT 29 ss and 30 ss embryos show the absence of an anterior stripe of expression (**A**, **C**) and presence of such a band (**B**, **D**) at the same somite stages. **Black arrow** indicates last somite boundary, **asterisks** indicate the 28^th^ somite, note relative position to vessels of the allantois to the last somite is also useful to stage embryos (**red arrows**).
(TIF)

S2 Fig *Fgf3* mutant clock oscillation is unaffected. Notch oscillations within the PSM were examined by *Hes7* mRNA expression in E10.5 controls (**A**) and *Fgf3* mutants (**B**) (33–41 ss). Measurements of each domain of PSM expression were taken as indicated by the black and white bars superimposed on the images and data is graphed below. Samples are organized by phase of oscillation. Note the relative distance between the rostral domain of *Hes7* expression and the last formed somite (indicated by top white bar) is noticeably smaller in *Fgf3* mutants, indicative of the formation of smaller somites.
(TIF)

S3 Fig Somite patterning is unaffected in *Fgf3* mutants. (**A**-**D**) WISH assays for *Pax3* (A, B) and *Pax1* (C, D) at 30 ss.
(TIF)

S4 Fig FGF-responsive gene expression is unaffected in the PSM of *Fgf3* mutants. WISH assays for indicated markers at 28 ss (**A**-**D**) and 36 ss (**E**-**H’**). Dotted lines (**E**, **F**, **G**, **H**) indicate relative position of corresponding transverse section (**E’**, **F’**, **G’**, **H’**). **Inset C,D**: 46 ss.
(TIF)

S5 Fig Cell proliferation is not significantly changed in *Fgf3* mutant mesoderm and compound *Fgf3; Bak; Bax* triple null homozygote neural tube. Quantification of phospho-histone H3 positive nuclei per total nuclei for *Fgf3* ^*Δ/Δ*^ mutants and *Fgf3* ^*Δ /wt*^ control tailbud (TB) and neural tube tissues (NT)(A), and neural tube tissue of *Fgf3* ^*Δ/Δ*^*; Bak* ^*Δ/Δ*^*; Bax* ^*Δ/Δ*^ mutants and *Fgf3* ^*Δ/Δ*^*; Bak* ^*Δ/Δ*^ controls (B)(n = 3 for each genotype, percentage represents positive nuclei per total nuclei). Error bars represent SEM, no significant differences found using two-tailed t-test; however, 26–28 ss *Fgf3* ^*Δ/Δ*^*; Bak* ^*Δ/Δ*^*; Bax* ^*Δ/Δ*^ mutants had a higher rate of proliferation trending towards significance (p = 0.13).
(TIF)

S6 Fig *Fgf8intron* labels mesoderm progenitor domain. (**A**) Dorsal view of *WISH* assay for nascent *Fgf8* transcript as detected by a probe specific to the fifth intronic region of the *Fgf8* coding region (*Fgf8intron*), showing expression limited to the region of the tailbud progenitors at E10.5. (**B**) Sagittal section through *Fgf8intron* E10.5 tailbud showing expression in the caudal neuroectoderm and adjacent mesoderm within the chordoneural hinge (**box**); also note domain in caudal tail gut (**asterisk**) (**NT**: neural tube, **No**: notochord, **GT**: gut tube). (**C**) Utilizing a probe for the coding regions of *Fgf8* (*Fgf8exon*) labels a majority of the PSM. (**D**) Diagram describing *Fgf8* expression: 1) Transcription takes place in the PSM progenitors 2) Cells are displaced from the progenitor domain and stop transcribing *Fgf8* however 3) *Fgf8* mRNA perdures in these cells distributing the message throughout the PSM.
(TIF)

S7 Fig Cell death is not required for normal axis extension termination. Homozygous deletion of *Bak* (*Bak* ^*Δ/Δ*^) has minimal effects on cell death in the tailbud at E12.5 and E13.5 (**A** and **B**, lysotracker red staining, lateral view). *Bak* and *Bax* double nulls (Bak ^*Δ/Δ*^*; Bax* ^*Δ/Δ*^) however have no detectable cell death in this domain (**D** and **E**) yet form the same number of caudal vertebrae as controls (**F** and **F’**, compare to **C** and **C’**, E18.5, numbers indicate the number of the caudal vertebra).
(TIF)

S8 Fig Compound *Fgf3; Bak; Bax* triple null homozygotes have *spina bifida* and *spina bifida occulta*. Homozygous deletion of *Bak* in *Fgf3* mutants (*Fgf3* ^*Δ/Δ*^*; Bak* ^*Δ/Δ*^) leads to normal spinal development (**A**, **C**, **E**). Homozygous deletion of both *Bak* and *Bax* in *Fgf3* mutants (*Fgf3* ^*Δ/Δ*^*; Bak* ^*Δ/Δ*^*; Bax* ^*Δ/Δ*^) causes *spina bifida* (**B**, **red arrow** points to opening in epithelium) and *spina bifida occulta* (**D**, **F**). **Yellow double-headed arrow** indicates open neural arches; **bracket** in **F** indicates abnormal gap between neural arches of sacral vertebra 2.
(TIF)

S9 Fig *Noggin* expression is unaffected in *Fgf3* mutants. Lateral view of WISH assay for *Noggin* expression shows normal expression in mutants (**C**, **D**) compared to controls (**A**, **B**).
(TIF)

S10 Fig Neural crest derived dorsal root ganglion patterning is unaffected in *Fgf3* mutants. Neurofilament1 (**NF1**) staining of dorsal root ganglion shows normal patterning in E11.5 *Fgf3* mutants (**B**) compared to littermate controls (**A**) at the A-P level of 26–34 somites; **yellow arrow** indicates posterior edge of hindlimb bud.
(TIF)

S11 Fig *Msx1* expression in the PSM during normal axis extension. Lateral view of WISH assay for *Msx1* in an *Fgf3*-heterozygous E11.5 (45 ss) embryos showing expression in the dorsal PSM (arrow).
(TIF)

S12 Fig Altering BMP signaling affects neural tube width. (**A**-**H**) WISH assays for indicated probes. Note that *Msx1* WISH in **A** and **B** (dorsal views, 28 ss) and **E** and **F** (lateral views, 30 ss) was performed for a relatively short period to reveal qualitative differences in intensity between genotypes. Transverse sections of 28 ss embryos of indicated genotype (**C**, **D**, **G**, **F**) are at the approximate anterior-position of the anterior PSM.
(TIF)

S13 Fig *Bmp4* expression is increased in *Fgf3; Bak; Bax* triple null homozygotes. *Fgf3* ^*Δ/Δ*^*; Bak* ^*Δ/Δ*^*; Bax* ^*Δ/Δ*^ triple null homozygotes (**B**, **D**) have an increase in *Bmp4* expression in the dorsal neural tube at 25ss (**B**) and 36ss (**D**) as compared to stage-matched littermate *Fgf3* ^*Δ/Δ*^ (**A**) and *Fgf3* ^*Δ/Δ*^*; Bax* ^*Δ/WT*^ controls (**C**). Boxes in **A** and **B** indicate area of image enlarged and placed above whole tail image; **red arrows** mark posterior extent of neural tube *Bmp4* expression. Migratory neural crest, marked by *FoxD3* expression, are caudally expanded in *Fgf3* ^*Δ/Δ*^*; Bak* ^*Δ/Δ*^*; Bax* ^*Δ/Δ*^ mutants (**arrowheads**, **F** and **F’**) as compared to littermate *Fgf3* ^*Δ/Δ*^*; Bak* ^*Δ/ Δ*^ controls (**arrowheads**, **E**, and **E’**). In triple mutants, the premigratory neural crest is expanded rostrally (**red asterisks)** although the caudal limit is similar to *Fgf3* ^*Δ/Δ*^*; Bak* ^*Δ/ Δ*^ littermate controls. (**dotted lines** mark anterior boundary of PSM).
(TIF)
