# Supplementary figures and images for: An FGF3-BMP Signaling Axis Regulates Caudal Neural Tube Closure, Neural Crest Specification and Anterior-Posterior Axis Extension
Source: PLoS Genet. 2016 May 4;12(5):e1006018. doi: 10.1371/journal.pgen.1006018 (PMC4856314; doi:10.1371/journal.pgen.1006018)

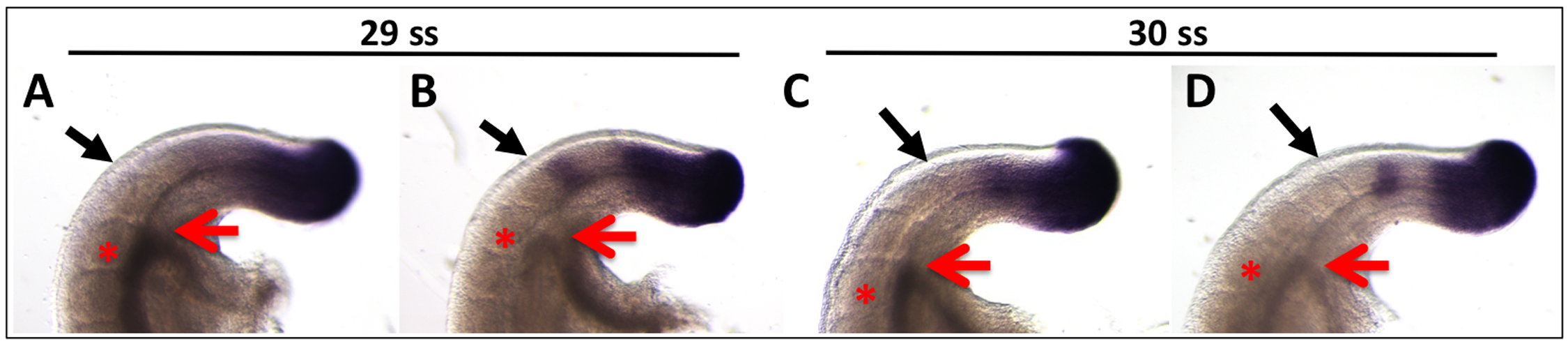

Supplement: S1 Fig — WISH assay for Fgf3 mRNA expression in WT 29 ss and 30 ss embryos show the absence of an anterior stripe of expression (A, C) and presence of such a band (B, D) at the same somite stages. Black arrow indicates last somite boundary, asterisks indicate the 28th somite, note relative position to vessels of the allantois to the last somite is also useful to stage embryos (red arrows). (TIF) [file pgen.1006018.s001.tif]

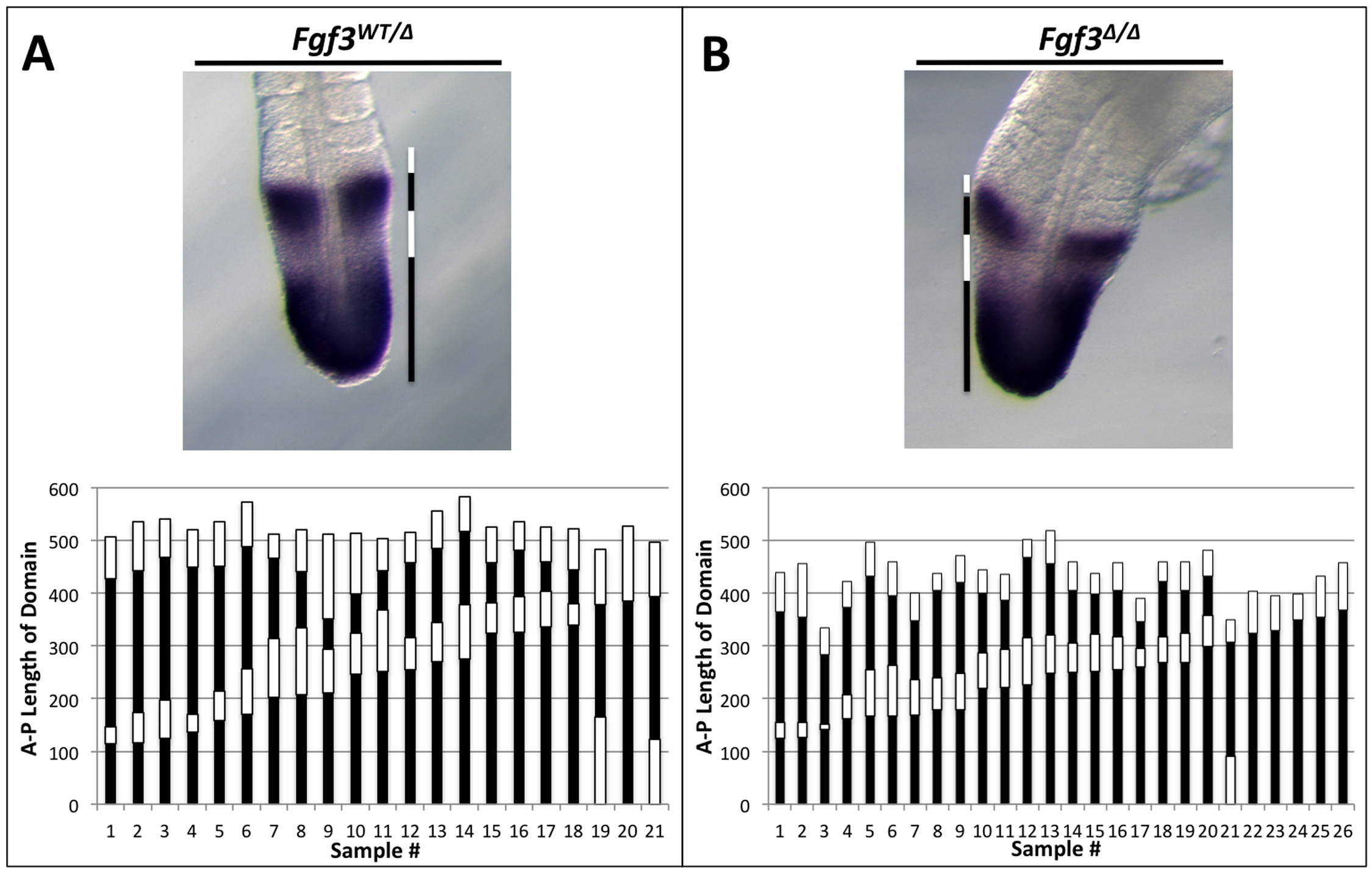

Supplement: S2 Fig — Notch oscillations within the PSM were examined by Hes7 mRNA expression in E10.5 controls (A) and Fgf3 mutants (B) (33–41 ss). Measurements of each domain of PSM expression were taken as indicated by the black and white bars superimposed on the images and data is graphed below. Samples are organized by phase of oscillation. Note the relative distance between the rostral domain of Hes7 expression and the last formed somite (indicated by top white bar) is noticeably smaller in Fgf3 mutants, indicative of the formation of smaller somites. (TIF) [file pgen.1006018.s002.tif]

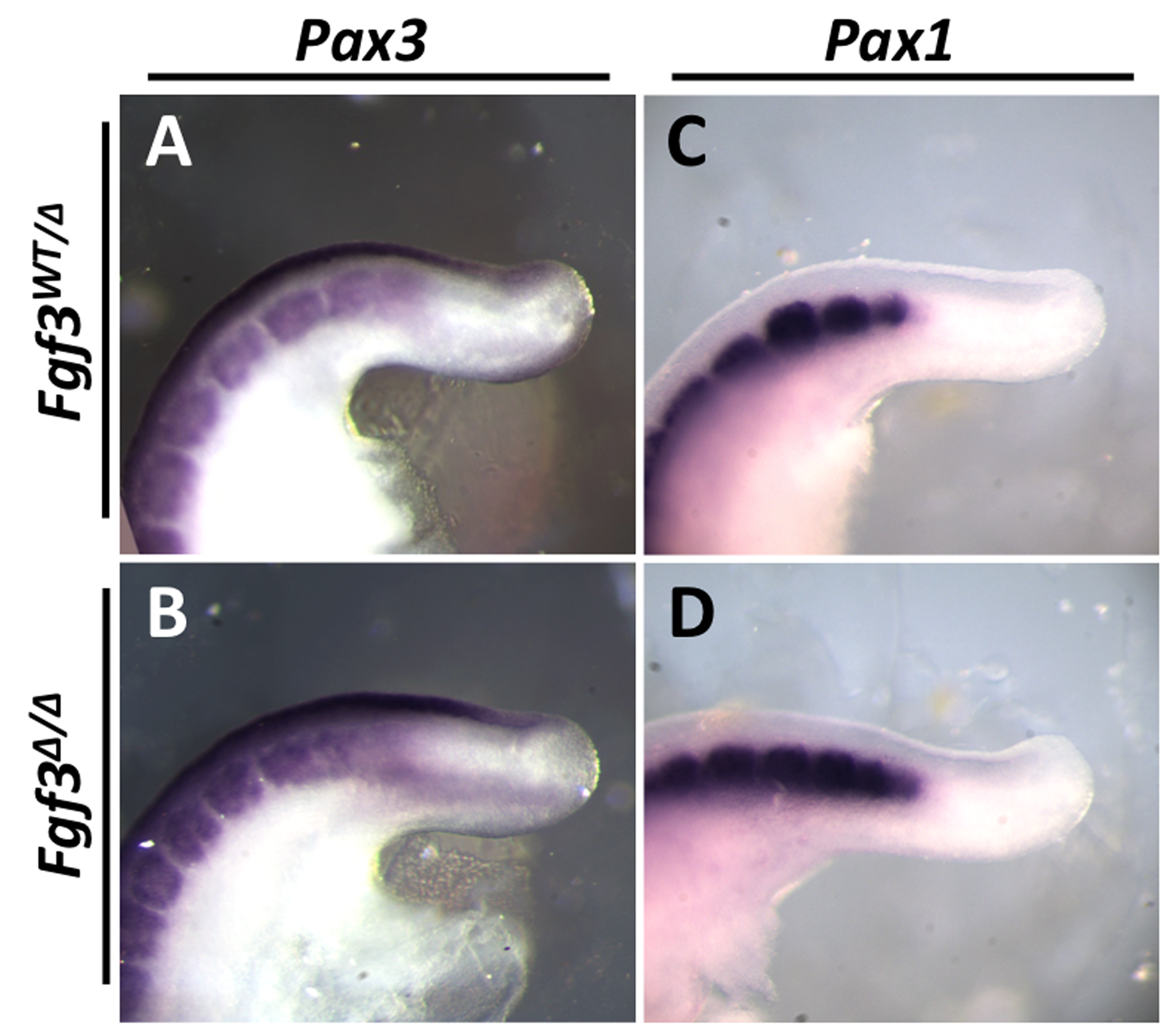

Supplement: S3 Fig — (A-D) WISH assays for Pax3 (A, B) and Pax1 (C, D) at 30 ss. (TIF) [file pgen.1006018.s003.tif]

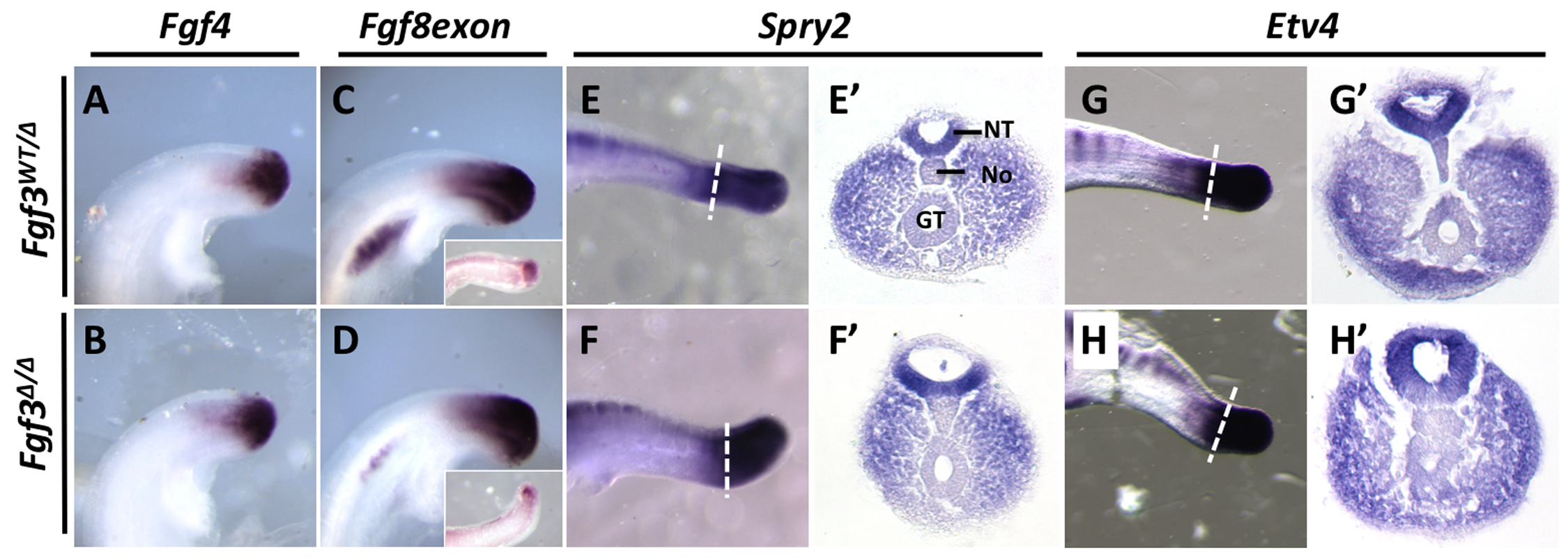

Supplement: S4 Fig — WISH assays for indicated markers at 28 ss (A-D) and 36 ss (E-H’). Dotted lines (E, F, G, H) indicate relative position of corresponding transverse section (E’, F’, G’, H’). Inset C,D: 46 ss. (TIF) [file pgen.1006018.s004.tif]

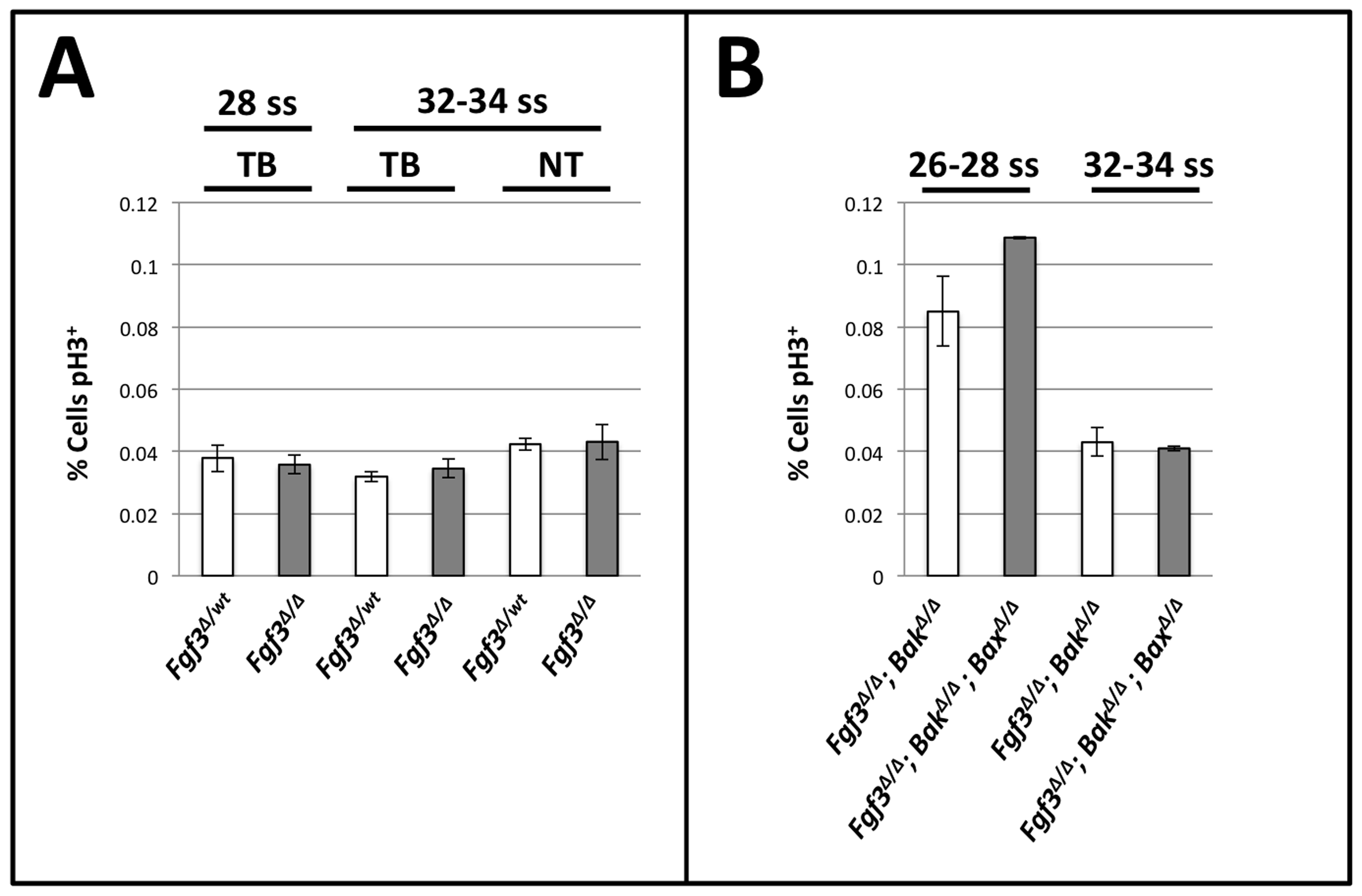

Supplement: S5 Fig — Quantification of phospho-histone H3 positive nuclei per total nuclei for Fgf3 Δ/Δ mutants and Fgf3 Δ /wt control tailbud (TB) and neural tube tissues (NT)(A), and neural tube tissue of Fgf3 Δ/Δ; Bak Δ/Δ; Bax Δ/Δ mutants and Fgf3 Δ/Δ; Bak Δ/Δ controls (B)(n = 3 for each genotype, percentage represents positive nuclei per total nuclei). Error bars represent SEM, no significant differences found using two-tailed t-test; however, 26–28 ss Fgf3 Δ/Δ; Bak Δ/Δ; Bax Δ/Δ mutants had a higher rate of proliferation trending towards significance (p = 0.13). (TIF) [file pgen.1006018.s005.tif]

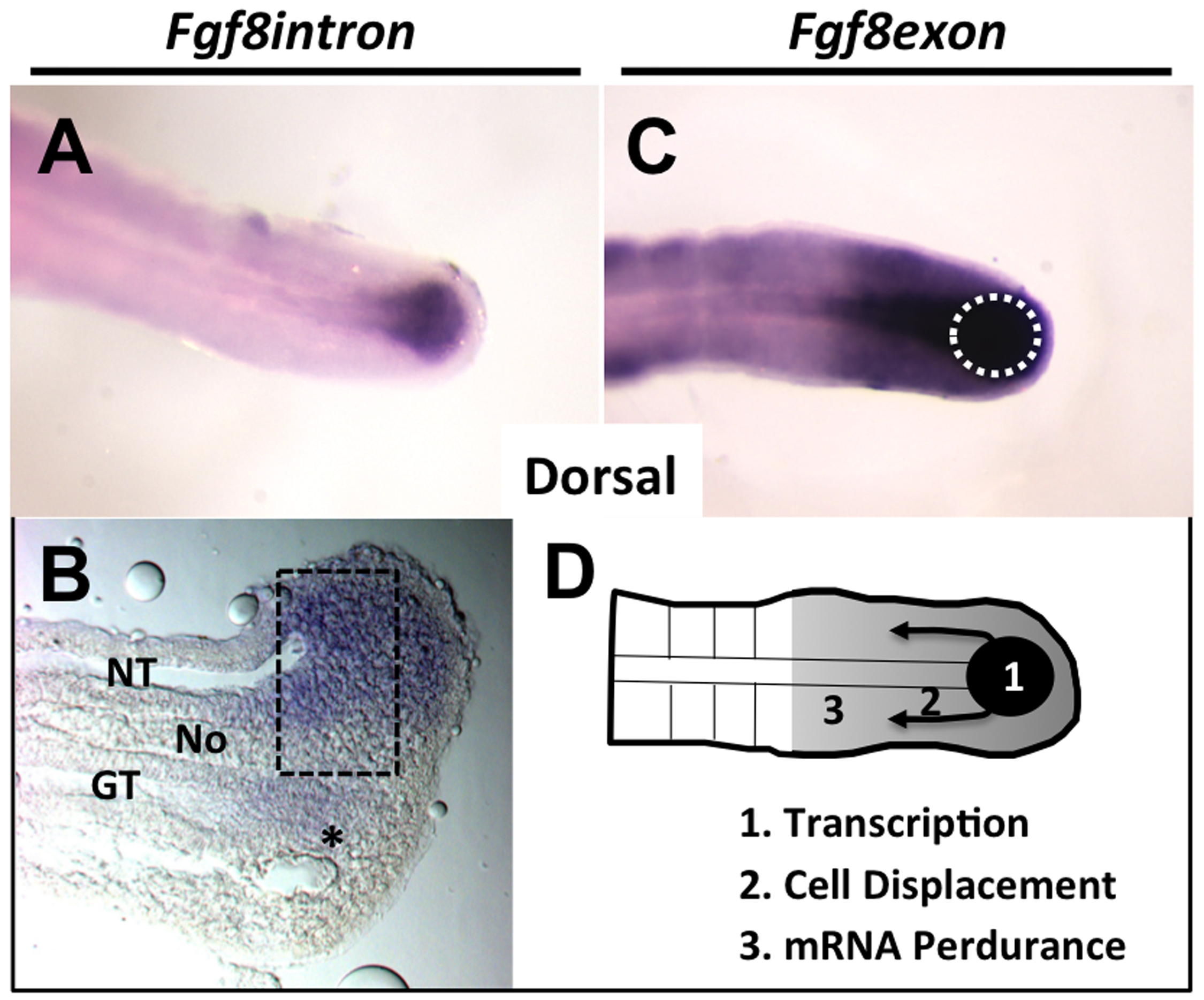

Supplement: S6 Fig — (A) Dorsal view of WISH assay for nascent Fgf8 transcript as detected by a probe specific to the fifth intronic region of the Fgf8 coding region (Fgf8intron), showing expression limited to the region of the tailbud progenitors at E10.5. (B) Sagittal section through Fgf8intron E10.5 tailbud showing expression in the caudal neuroectoderm and adjacent mesoderm within the chordoneural hinge (box); also note domain in caudal tail gut (asterisk) (NT: neural tube, No: notochord, GT: gut tube). (C) Utilizing a probe for the coding regions of Fgf8 (Fgf8exon) labels a majority of the PSM. (D) Diagram describing Fgf8 expression: 1) Transcription takes place in the PSM progenitors 2) Cells are displaced from the progenitor domain and stop transcribing Fgf8 however 3) Fgf8 mRNA perdures in these cells distributing the message throughout the PSM. (TIF) [file pgen.1006018.s006.tif]

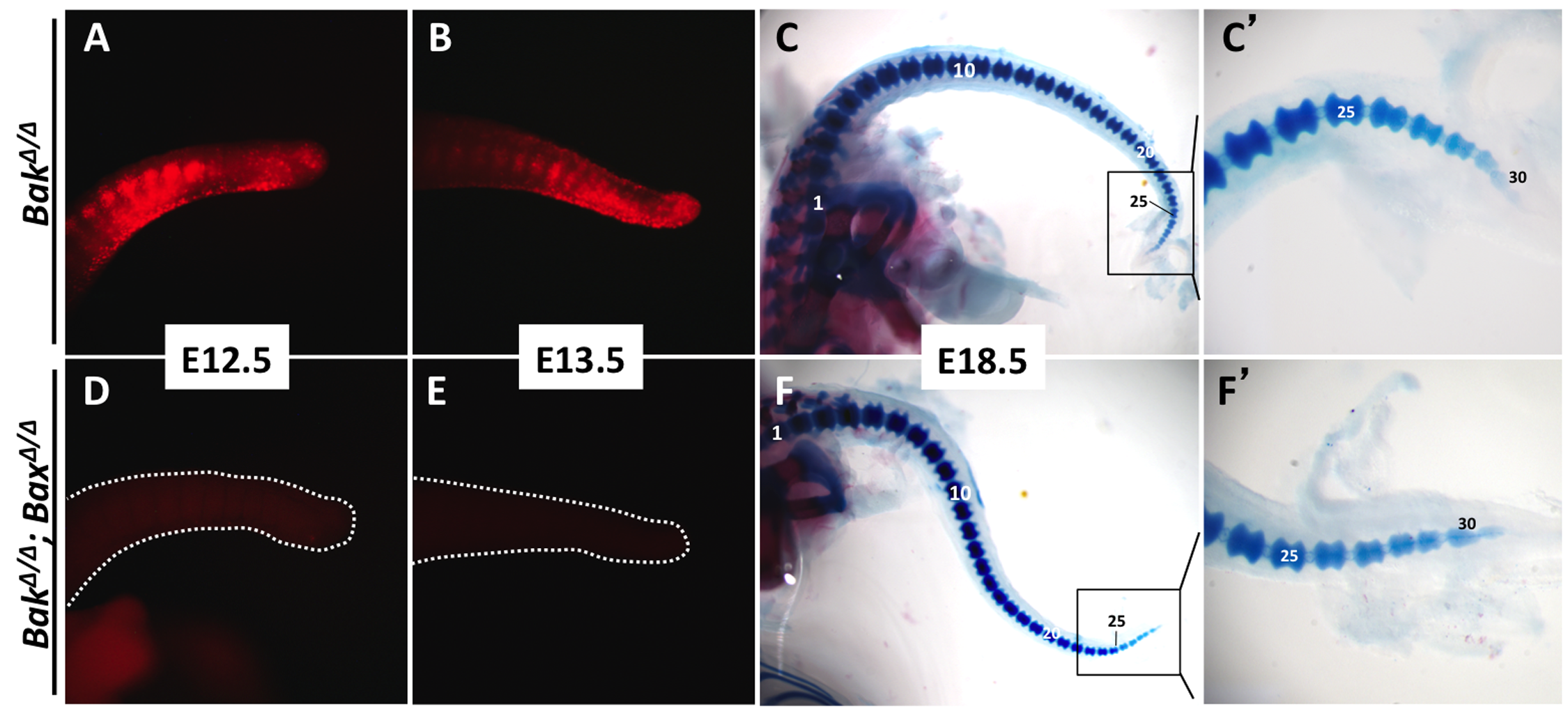

Supplement: S7 Fig — Homozygous deletion of Bak (Bak Δ/Δ) has minimal effects on cell death in the tailbud at E12.5 and E13.5 (A and B, lysotracker red staining, lateral view). Bak and Bax double nulls (Bak Δ/Δ; Bax Δ/Δ) however have no detectable cell death in this domain (D and E) yet form the same number of caudal vertebrae as controls (F and F’, compare to C and C’, E18.5, numbers indicate the number of the caudal vertebra). (TIF) [file pgen.1006018.s007.tif]

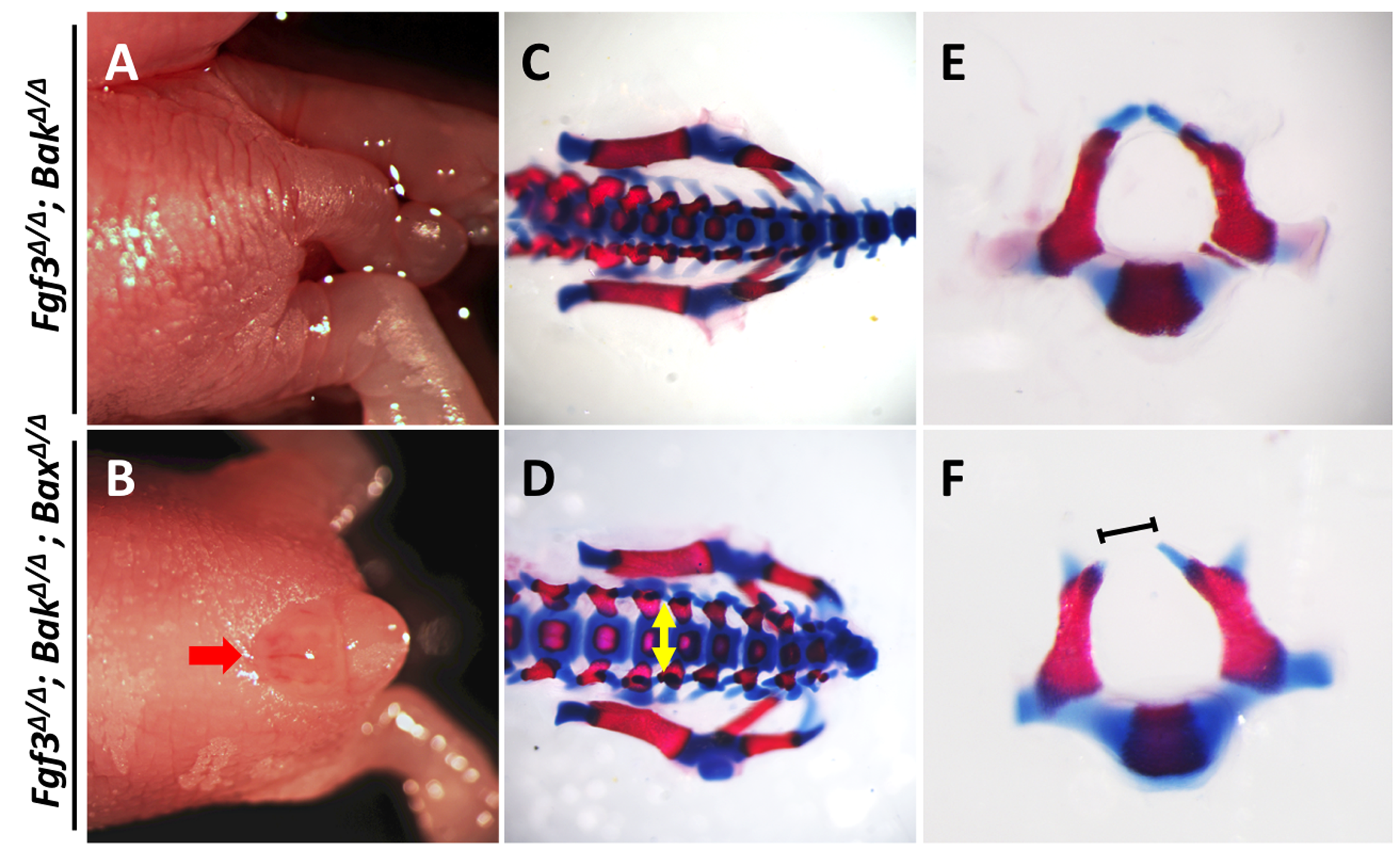

Supplement: S8 Fig — Homozygous deletion of Bak in Fgf3 mutants (Fgf3 Δ/Δ; Bak Δ/Δ) leads to normal spinal development (A, C, E). Homozygous deletion of both Bak and Bax in Fgf3 mutants (Fgf3 Δ/Δ; Bak Δ/Δ; Bax Δ/Δ) causes spina bifida (B, red arrow points to opening in epithelium) and spina bifida occulta (D, F). Yellow double-headed arrow indicates open neural arches; bracket in F indicates abnormal gap between neural arches of sacral vertebra 2. (TIF) [file pgen.1006018.s008.tif]

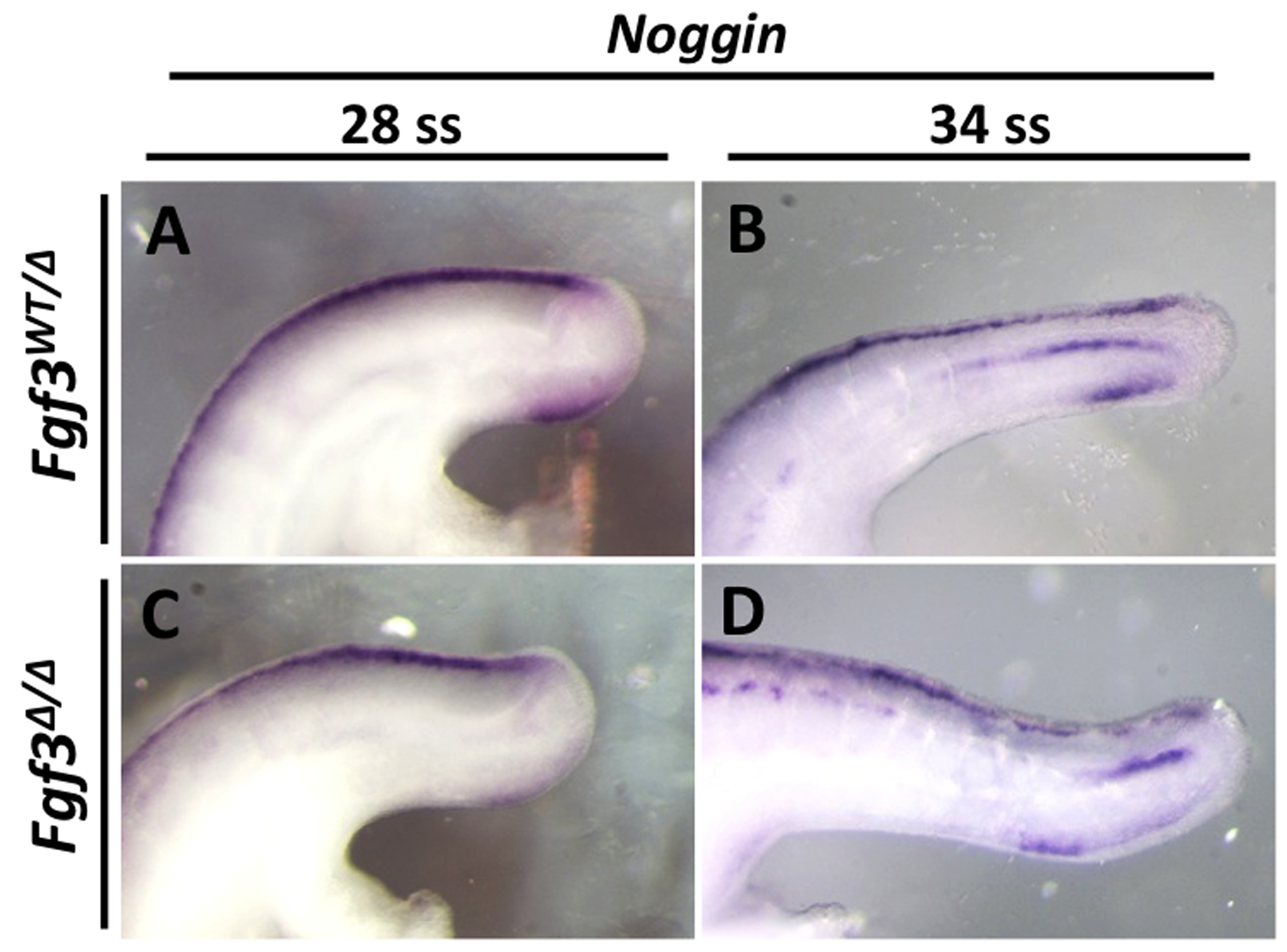

Supplement: S9 Fig — Lateral view of WISH assay for Noggin expression shows normal expression in mutants (C, D) compared to controls (A, B). (TIF) [file pgen.1006018.s009.tif]

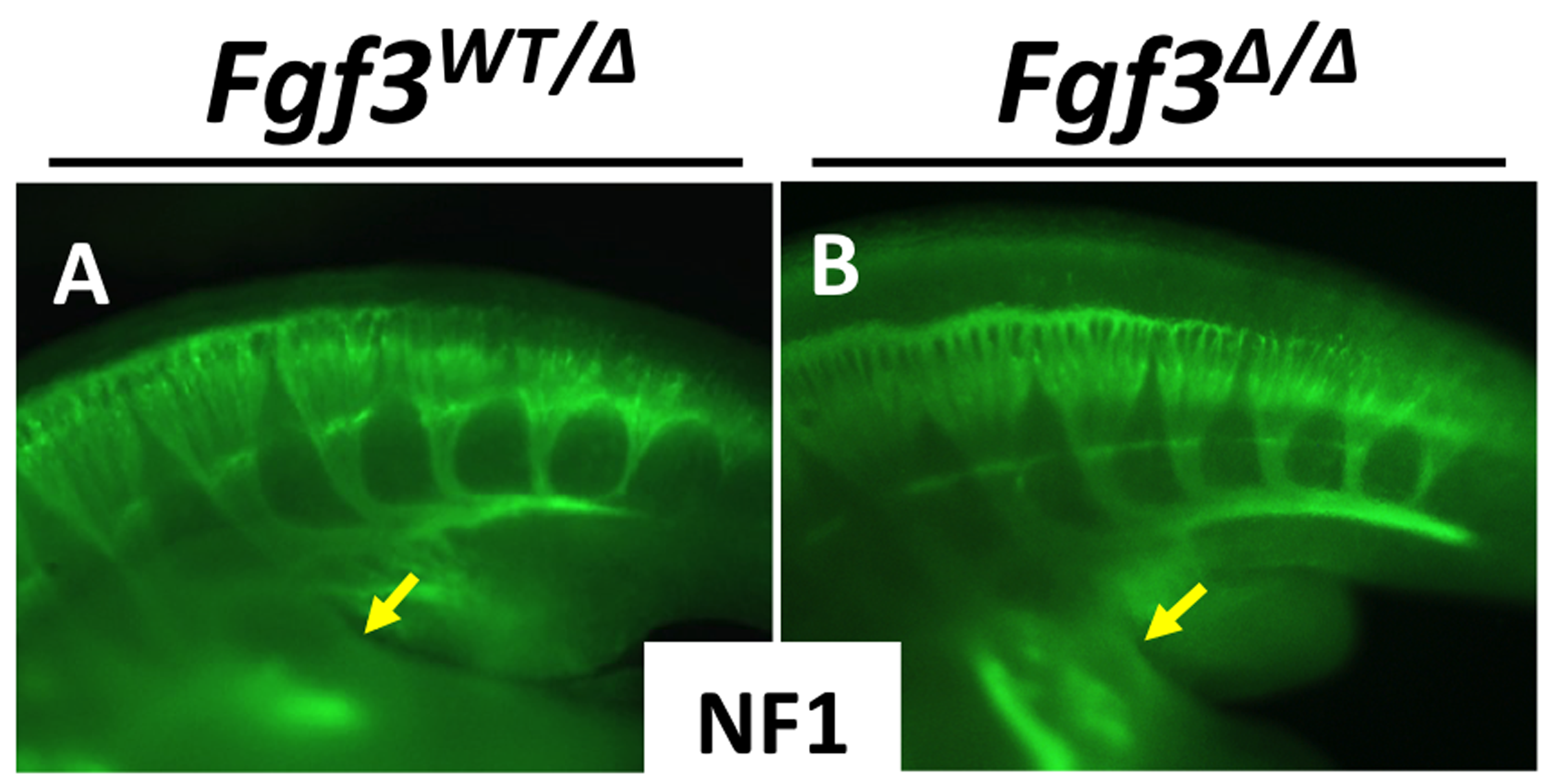

Supplement: S10 Fig — Neurofilament1 (NF1) staining of dorsal root ganglion shows normal patterning in E11.5 Fgf3 mutants (B) compared to littermate controls (A) at the A-P level of 26–34 somites; yellow arrow indicates posterior edge of hindlimb bud. (TIF) [file pgen.1006018.s010.tif]

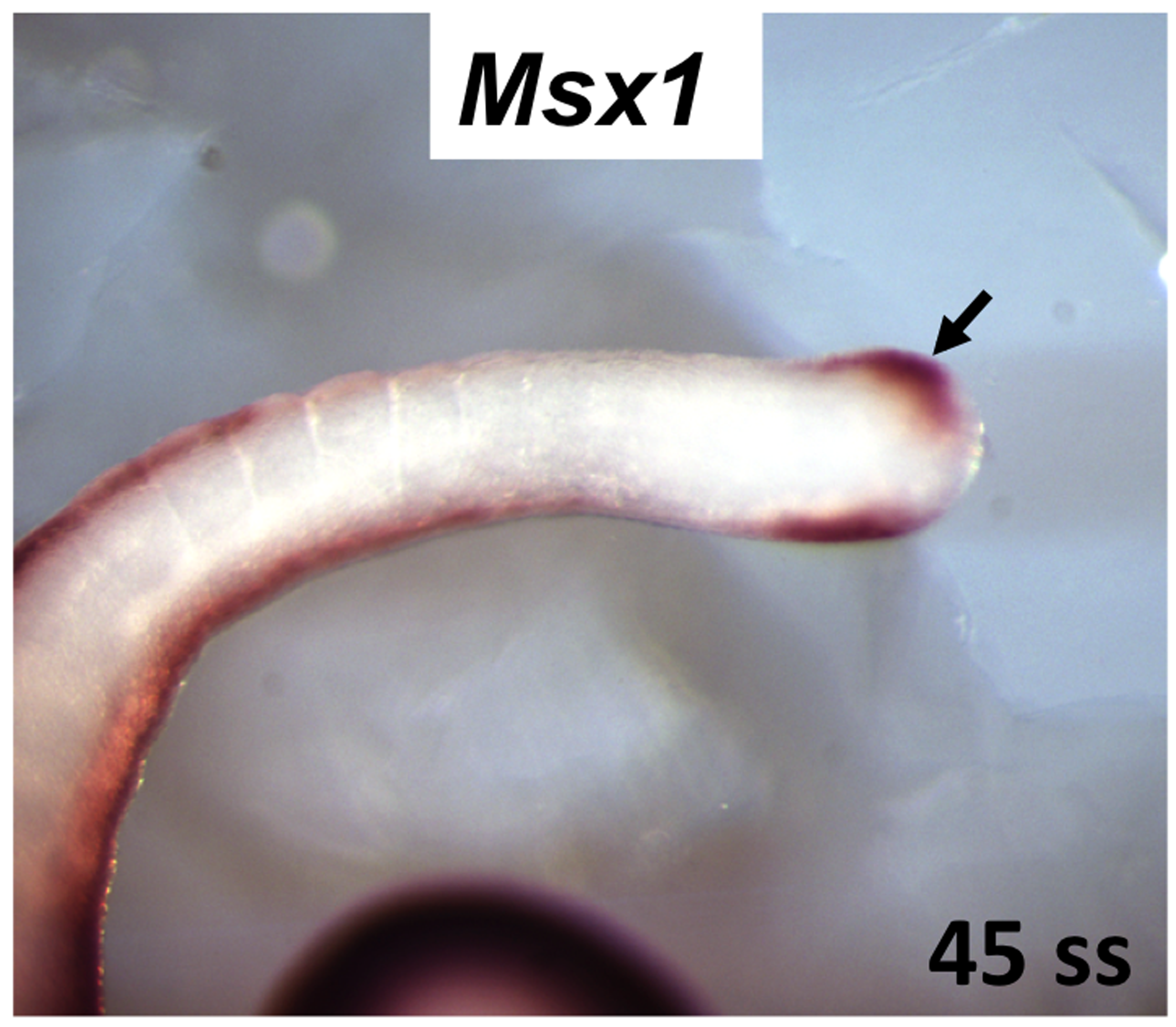

Supplement: S11 Fig — Lateral view of WISH assay for Msx1 in an Fgf3-heterozygous E11.5 (45 ss) embryos showing expression in the dorsal PSM (arrow). (TIF) [file pgen.1006018.s011.tif]

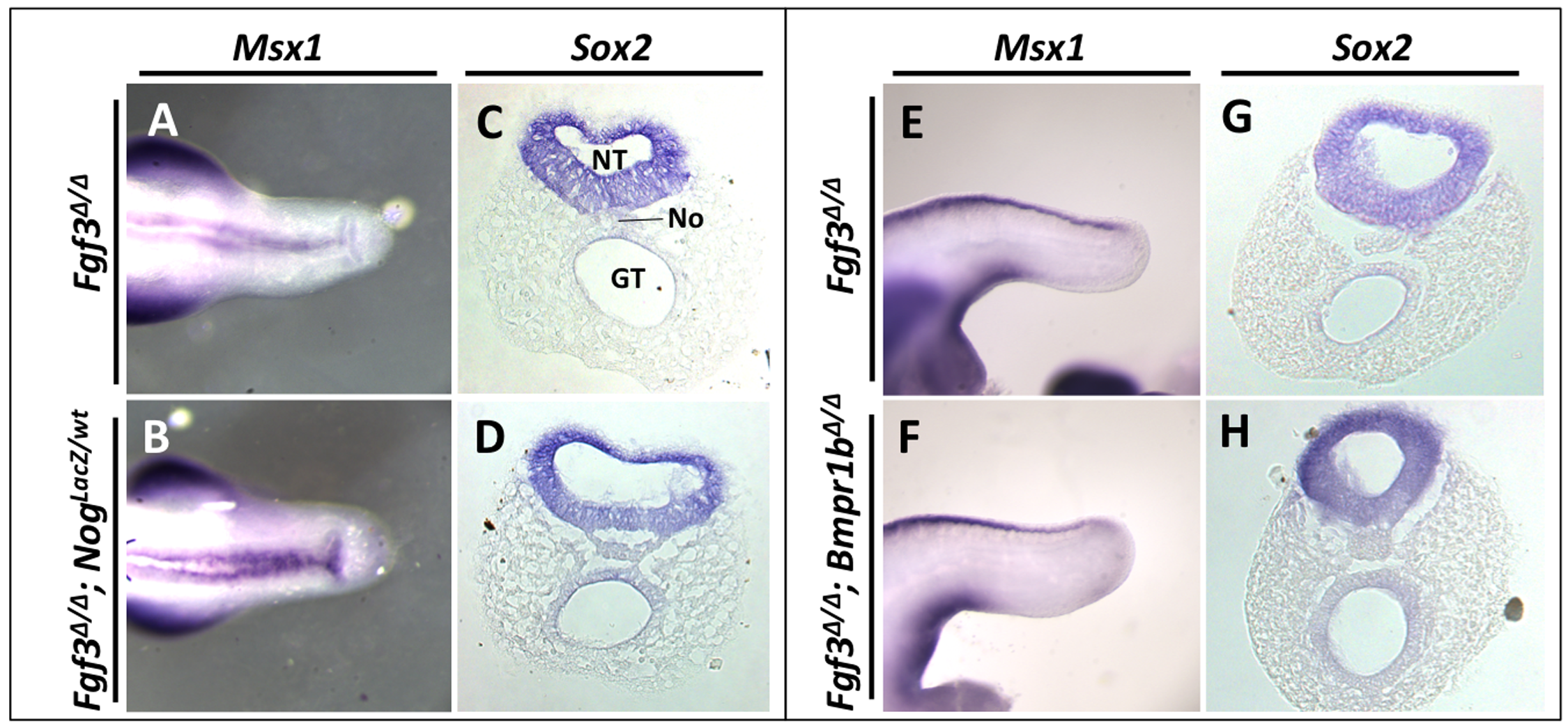

Supplement: S12 Fig — (A-H) WISH assays for indicated probes. Note that Msx1 WISH in A and B (dorsal views, 28 ss) and E and F (lateral views, 30 ss) was performed for a relatively short period to reveal qualitative differences in intensity between genotypes. Transverse sections of 28 ss embryos of indicated genotype (C, D, G, F) are at the approximate anterior-position of the anterior PSM. (TIF) [file pgen.1006018.s012.tif]

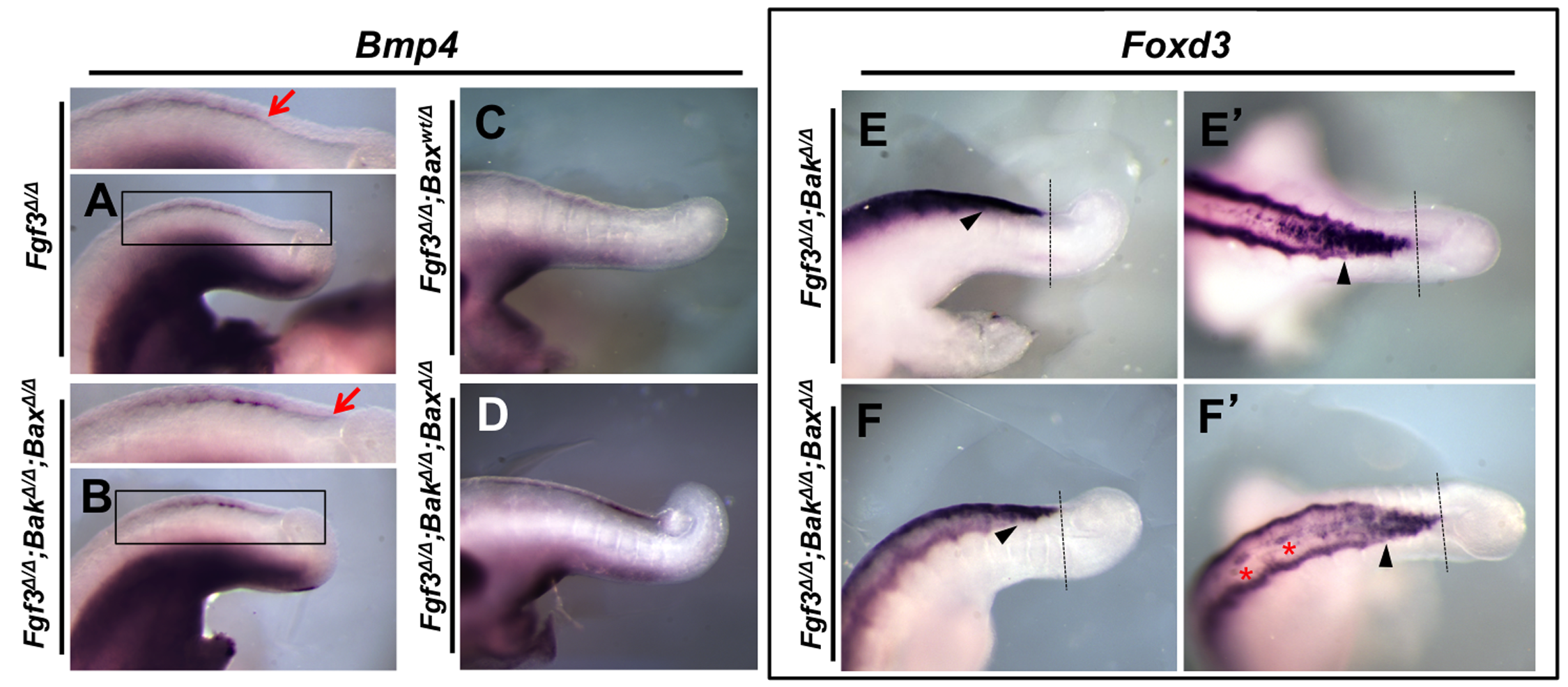

Supplement: S13 Fig — Fgf3 Δ/Δ; Bak Δ/Δ; Bax Δ/Δ triple null homozygotes (B, D) have an increase in Bmp4 expression in the dorsal neural tube at 25ss (B) and 36ss (D) as compared to stage-matched littermate Fgf3 Δ/Δ (A) and Fgf3 Δ/Δ; Bax Δ/WT controls (C). Boxes in A and B indicate area of image enlarged and placed above whole tail image; red arrows mark posterior extent of neural tube Bmp4 expression. Migratory neural crest, marked by FoxD3 expression, are caudally expanded in Fgf3 Δ/Δ; Bak Δ/Δ; Bax Δ/Δ mutants (arrowheads, F and F’) as compared to littermate Fgf3 Δ/Δ; Bak Δ/ Δ controls (arrowheads, E, and E’). In triple mutants, the premigratory neural crest is expanded rostrally (red asterisks) although the caudal limit is similar to Fgf3 Δ/Δ; Bak Δ/ Δ littermate controls. (dotted lines mark anterior boundary of PSM). (TIF) [file pgen.1006018.s013.tif]
